# Biogenic nanosilver bearing antimicrobial and antibiofilm activities and its potential for application in agriculture and industry

**DOI:** 10.3389/fmicb.2023.1125685

**Published:** 2023-02-20

**Authors:** Joanna Trzcińska-Wencel, Magdalena Wypij, Mahendra Rai, Patrycja Golińska

**Affiliations:** ^1^Department of Microbiology, Nicolaus Copernicus University in Toruń, Toruń, Poland; ^2^Nanobiotechnology Laboratory, Department of Biotechnology, SGB Amravati University, Amravati, Maharashtra, India

**Keywords:** AgNPs, biofilm, food-borne pathogens, human pathogens, mycosynthesis, plant pathogens

## Abstract

**Introduction:**

Due to the increasing resistance of bacteria and fungi to antimicrobials, it is necessary to search for effective alternatives to prevent and treat pathogens causing diseases in humans, animals, and plants. In this context, the mycosynthesized silver nanoparticles (AgNPs) are considered as a potential tool to combat such pathogenic microorganisms.

**Methods:**

AgNPs were synthesized from *Fusarium culmorum* strain JTW1 and characterized by Transmission Electron Microscopy (TEM), X-ray diffraction (XRD), Fourier Transform Infrared (FTIR) spectroscopy, Nanoparticle Tracking Analysis (NTA), Dynamic Light Scattering (DLS) and Zeta potential measurement. The minimum inhibitory (MIC) and biocidal concentrations (MBC) were determined against 13 bacterial strains. Moreover, the combined effect of AgNPs with antibiotics (streptomycin, kanamycin, ampicillin, tetracycline) was also studied by determining the Fractional Inhibitory Concentration (FIC) index. The anti-biofilm activity was examined by crystal violet and fluorescein diacetate (FDA) assays. Furthermore, antifungal activity of AgNPs was evaluated against a panel of phytopathogenic fungi *viz.*, *Botrytis*, *Colletotrichum*, *Fusarium*, *Phoma*, *Sclerotinia*, and an oomycete pathogen *Phytophthora* by agar well-diffusion and micro-broth dilution method to evaluate the minimal AgNPs concentrations that inhibit fungal spore germination.

**Results:**

Fungi-mediated synthesis resulted in the formation of small (15.56 ± 9.22 nm), spherical and stable (zeta potential of – 38.43 mV) AgNPs with good crystallinity. The results of FTIR spectroscopy indicated the presence of various functional groups, namely hydroxyl, amino, and carboxyl ones, from the biomolecules on the surface of AgNPs. The AgNPs showed antimicrobial and antibiofilm formation activities against Gram-positive and Gram-negative bacteria. The values of MIC and MBC ranged between 16–64 and 32–512 μg mL^−1^, respectively. The enhanced effect of AgNPs in combination with antibiotics was confirmed against human pathogens. The highest synergistic effect (FIC = 0.0625) was demonstrated by the combination of AgNPs with streptomycin against two strains of *Escherichia coli* (ATCC 25922 and ATCC 8739), followed by *Klebsiella pneumoniae* and *Pseudomonas aeruginosa* (FIC = 0.125). Enhanced effects of AgNPs with ampicillin were also shown against *Staphylococcus aureus* ATCC 25923 (FIC = 0.125) and *P. aeruginosa* (FIC = 0.25), as well as kanamycin against *S. aureus* ATCC 6538 (FIC = 0.25). The crystal violet assay revealed that the lowest concentration of AgNPs (0.125 μg mL^−1^) reduced the development of biofilms of *Listeria monocytogenes* and *Salmonella enterica*, while the maximum resistance was shown by *Salmonella infantis*, its biofilm was reduced after exposure to a concentration of 512 μg mL^−1^. A high inhibitory effect on the activity of bacterial hydrolases was observed by the FDA assay. AgNPs at a concentration of 0.125 μg mL^−1^ reduced the hydrolytic activity of all biofilms formed by the tested pathogens, except *E. coli* ATCC 25922, *P. aeruginosa*, and *Pectobacterium carotovorum* (efficient concentration was 2-fold higher, at 0.25 μg mL^−1^), while the hydrolytic activity of *E. coli* ATCC 8739, *Salmonella infantis* and *S. aureus* ATCC 6538 was suppressed after treatment with AgNPs at concentrations of 0.5, 2 and 8 μg mL^−1^, respectively. Moreover, AgNPs inhibited fungal growth and spore germination of *Botrytis cinerea*, *Phoma lingam,* and *Sclerotinia sclerotiorum*. MIC and MFC values of AgNPs against spores of these fungal strains were determined at 64, 256, and 32 μg mL^−1^, and zones of growth inhibition were 4.93, 9.54, and 3.41 mm, respectively.

**Discussion:**

*Fusarium culmorum* strain JTW1 was found to be an eco-friendly biological system for an easy, efficient and inexpensive synthesis of AgNPs. In our study, the mycosynthesised AgNPs demonstrated remarkable antimicrobial (antibacterial and antifungal) and antibiofilm activities against a wide range of human and plant pathogenic bacteria and fungi singly and in combination with antibiotics. These AgNPs could be applied in medicine, agriculture, and food industry to control such pathogens that cause numerous human diseases and crop losses. However, before using them extensive animal studies are required to evaluate the toxicity, if any.

## 1. Introduction

Modern medicine, veterinary, food, and agriculture sectors are struggling with microbial diseases due to the increasing resistance of microorganisms to available antimicrobials agents (Food and Agriculture Organization of the United Nations (FAO), 2017; Xie et al., 2018; Hashempour-Baltork et al., 2019). Antibiotic resistance results from the excessive and reckless use of antibiotics in agricultural food production, healthcare, and environmental protection sectors (Xie et al., 2018; Caniça et al., 2019; Dadgostar, 2019; Larsson and Flach, 2022). A wide range of pathogenic bacteria cause chronic infections by forming complex multicellular structures known as biofilms. Moreover, some studies indicate that conventional antibiotics may induce phenotypic changes in bacterial cells which subsequently trigger biofilm formation (Olivares et al., 2020). Biofilms provide a stable protective environment for the dissemination of microorganisms due to a self-synthesized structure which is an organic and highly hydrated matrix composed of exopolysaccharides, proteins, and nucleic acids (Hall-Stoodley and Stoodley, 2005; Olivares et al., 2020). There are several hypotheses of biofilm recalcitrant to antibiotics, including persistent cells, adaptive responses, and lower penetration of antimicrobial agents (Sahoo et al., 2021). In response to the growing threat of bacterial pathogens, it is necessary to develop approaches that are wide-ranging and effective in controlling bacterial outbreaks. An alternative to conventional chemicals is the formulation of agents that may prevent biofilm formation and also act on individual bacterial cells.

The growing population and increasing demand for food, require solutions to increase crop yields and ensure safety in food production, distribution, and storage (Fung et al., 2018; Wypij et al., 2023). Nowadays, microbial diseases of plants are becoming an increasing problem as they reduce crop yields and significantly affect the food industry on both a global and regional scale (Charkowski, 2018; Charkowski et al., 2020). Among the most notable bacterial pathogens of plants are *Pseudomonas syringae*, *Ralstonia solanacearum, Agrobacterium tumefaciens, Xanthomonas* spp., *Erwinia amylovora,* and *Pectobacterium carotovorum* (Mansfield et al., 2012; Iwu and Okoh, 2019). The bacterial diseases of crop plants including black rot (Schaad et al., 1980; Maji and Nath, 2015), soft rot (Pérombelon and Kelman, 1980; Pérombelon, 2002), bacterial speck (Shenge et al., 2007; Butsenko et al., 2020), and fire blight (Johnson and Stockwell, 1998; Vanneste, 2000; Peil et al., 2021). While, fungal pathogens, for example, *Fusarium oxysporum, Botrytis cinerea, Colletotrichum acutatum, Puccinia* sp.*, Sclerotinia sclerotiorum,* etc., have a broad host range and cause pre-and post-harvest diseases. Among them, causal agents of grey and white mold as well as black leg and dry rot are worthy of note (Amselem et al., 2011; Dean et al., 2012; Gabal et al., 2019). Plant pathogens spread rapidly and are increasingly aggressive and harmful leading to yield losses in cereals (maize, wheat, rice), vegetables (potatoes, tomatoes, brassicas), and fruits (Sundin et al., 2016; Nawaz et al., 2020; Hampf et al., 2021). Indeed, foodborne illnesses caused by bacteria, are another concern with ensuring human food security (Gandhi and Chikindas, 2007; Scallan Walter et al., 2021). Bacterial pathogens such as *Listeria* sp., or the the members of *Enterobacteriaceae* family are proliferated *via* the fecal-oral routes by ingestion of contaminated water or food. Virulent strains of these bacteria cause a broad-spectrum of disorders like nausea, watery or bloody diarrhea, vomiting, and inflammatory changes (Allocati et al., 2013). Thus, intracellular pathogen *Listeria monocytogenes* is responsible for listeriosis, a very severe and deadly illness (Chlebicz and Śliżewska, 2018). Symptoms of the infection in healthy adult patients may include lack of appetite, stomachache, nausea, or diarrhea. Nonetheless, the infection is particularly dangerous for newborns, the elderly, immunocompromised patients, and poses a risk of miscarriage for pregnant women (Zhu et al., 2017).

Silver nanoparticles (AgNPs) have pronounced antimicrobial activity, even at low concentrations (Salleh et al., 2020). Some studies indicate, that AgNPs reveal long-term antibiofilm activities by multi-site action due to their unique properties, which include nano-size and high surface area (Martinez-Gutierrez et al., 2013; Ali et al., 2015; Soliman et al., 2022). Basically, biological methods of nanoparticle synthesis have been recognized as a substitute for physical and chemical ones (Rai et al., 2021b; Al-Rajhi et al., 2022). In addition, biologically synthesized AgNPs are capped with molecules of natural origin, which enables them to interact easily with bactericides along with bacterial cells and therefore, improves their antimicrobial efficiency (Wypij et al., 2020). Among the biological synthesis methods of nanoparticles (NPs) are those mediated by plants, algae, bacteria or fungi (Wypij et al., 2021; Al-Rajhi et al., 2022; Salem et al., 2022, 2023). Among microorganisms, fungi reveal the strong potential to secrete metabolites involved in the synthesis of metallic nanoparticles (MNPs) (Rai et al., 2021a; Shaheen et al., 2021). Mycosynthesis is an environmentally friendly, inexpensive, and simple method, which can be used for the efficient production of AgNPs (Lahiri et al., 2021; Salem et al., 2023). Current research efforts focus on the optimization of mycosynthesis of AgNPs with desired physio-chemical and biological properties, which may be used to eradicate bacterial pathogens (Zhao et al., 2018).

Hence, the present study was designed to synthesize AgNPs from *Fusarium culmorum* strain JTW1 in an easy, eco-friendly, efficient, and inexpensive way and to estimate the potential use of AgNPs in the biomedicine, agriculture, and food production industry. The mycosynthesized AgNPs were characterized using UV–vis spectrophotometry (UV–Vis), Transmission Electron Microscopy (TEM), X-ray diffraction (XRD) spectroscopy, Energy-dispersive X-ray spectroscopy (EDX), Fourier-Transform Infrared Spectroscopy (FTIR), Nanoparticle Tracking Analysis (NTA), Dynamic Light Scattering (DLS) and Zeta potential measurement. The comprehensive antibacterial activity of AgNPs, including estimation of minimal inhibitory and biocidal concentrations (MIC and MBC, respectively), efficacy of combined antibiotics and AgNPs as well as antibiofilm activity against a wide range of Gram-positive and Gram-negative pathogenic bacteria of humans and plants were investigated. Mycosynthesized AgNPs were tested against a set of fungal and oomycete plant pathogens, including *Alternaria alternata* IOR 1783, *Botrytis cinerea* IOR 1873, *Colletotrichum acutatum* IOR 2153, *Fusarium oxysporum* IOR 342, *Fusarium solani* IOR 825, *Phoma lingam* IOR 2284, *Sclerotinia sclerotiorum* IOR 2242, *Phytophthora cactorum* IOR 1925, *Phytophthora cryptogea* IOR 2080, *Phytophthora megasperma* IOR 404 and *Phytophthora plurivora* IOR 2303.

## 2. Materials and methods

### 2.1. Isolation and identification

*Fusarium culmorum* strain JTW1 was isolated using ten-fold dilution procedure on Potato Dextrose Agar (PDA, A&A Biotechnology) from soil samples collected in Różankowo near Toruń, Poland. The Petri plates were incubated at 26°C for 7 days. Initially, the morphological and cultural characteristics of isolate *F. culmorum* strain JTW1 were studied after 7 days at 26°C on PDA. The isolate was identified by internal transcribed spacer (ITS) sequence. The genomic DNA of the isolate was extracted using Genomic Mini AX Yeast Spin Kit (A&A Biotechnology) according to the manufacturer’s instructions by A&A Biotechnology (Gdańsk, Poland) while amplification of ITS region and sequencing were carried out by Genomed S.A. (Warsaw, Poland). The ITS region of the ribosomal DNA of the isolate was amplified using the ITS1 and ITS2 primers (White et al., 1990). The Basic Local Alignment Search Tool (BLAST) at the National Centre of Biological Information (NCBI) was used to find the closest similarity between the isolate sequence and corresponding sequences available in the database. *F. culmorum* isolate JTW1 was deposited in the Deutsche Sammlung von Mikroorganismen und Zellkulturen (DSMZ) in Brunschweig, Germany under accession number DSM 114849.

### 2.2. Synthesis and characterization of mycosynthesized nanoparticles

*Fusarium culmorum* strain JTW1 was cultured in 250 ml Potato Dextrose Broth (PDB, A&A Biotechnology) for 7 days at 26°C in shaking conditions (120 revolutions per minute; rpm) and then the biomass was centrifugated at 6,500× *g* (Thermo Scientific, USA), washed three times with sterile distilled water, and re-suspended in sterile water for 4 days for cell autolysis. Thereafter, the cell filtrate was obtained by centrifugation of autolysate at 4000× *g* for 5 min and passing it through sterile Whatman filter paper No. 1. For mycosynthesis of AgNPs, the fungal extract obtained from *F. culmorum* strain JTW1 was treated with a 100 mM aqueous solution of silver nitrate (AgNO_3_; 1 mM final concentration). A biosynthesis reaction was induced on sunlight for 1 h and then the reaction mixture was incubated at room temperature in the dark. AgNPs were collected by centrifugation at 13,000× *g* (Thermo Scientific, USA) for 1 h and dried at 37°C (Thermo Scientific, USA; Wypij et al., 2022).

The physico-chemical analyses were performed as previously described by Wypij et al. (2018, 2022).

The primary detection of AgNPs was carried out by visual observation of color change after treatment of fungal extract with AgNO_3_ and sunlight induction. The UV–Visible (UV–Vis) spectrophotometer (NanoDrop 2000, Thermo Scientific, USA) was used for scanning the absorbance spectra in a range of wavelengths from 200 to 800 nm, at the resolution of 1 nm.

Transmission Electron Microscopy (TEM) and Energy Dispersive X-Ray (EDX) analyses were performed to determine the size, morphology, and elemental composition of AgNPs from *F. culmorum* strain JTW1. Prior to analysis, AgNPs solution was deposited on a carbon-coated copper grid (400 μm mesh size) and dried at room temperature. Analysis was performed using a transmission electron microscope (FEI Tecnai F20 X-Twintool, Fei, Hillsboro, OR, USA) operating at an acceleration voltage of 100 kV.

The crystalline nature of AgNPs was confirmed by X-ray diffraction analysis using Philips X’Pert diffractometer (X’Pert Pro, Analytical Philips, Lelyweg, Netherlands) equipped with Cu Kα (*λ* = 1.54056 Å) radiation source and using Ni as a filter in range 5°–120°. The collected peaks were compared to the standard database of the International Centre for Diffraction Data (ICDD).

The functional groups present on the surface of AgNPs were identified using Fourier-Transform Infrared (FTIR) spectroscopy. Briefly, a sample for analysis was prepared by combining dry AgNPs with KBr powder in a ratio of 1:100. The AgNPs were characterized by FTIR spectrophotometer (Perkin-Elmer FTIR-2000, USA) in the range 400–4,000 cm^−1^ at a resolution of 4 cm^−1^.

The size of synthesized nanoparticles was measured using Nanosight (NanoSight NS300, Malvern, UK). The solution of AgNPs in MilliQ water was sonicated at 20 Hz for 15 min (Sonic Ruptor 250, Omni Int., Kennesaw, GA, USA), 1000-fold diluted with the MilliQ water, and filtered through a 0.22-μm filter (Millipore) prior to analysis. During measurement, five 1-min videos were captured at the cell temperature of 25°C and syringe speed of 50 μl/s. The size distribution of AgNPs was analyzed using NanoSight Software NTA version 3.4 Build 3.4.4.

To determine the size distribution and zeta potential of AgNPs the Zetasizer Nano Instrument (Malvern Instruments Ltd., Malvern, United Kingdom) was used. The AgNPs size and dispersity were measured by dynamic light scattering (DLS) to obtain information about volume [%] as a function of particle size [nm]. Zeta potential measurement and nanoparticle size distribution analysis were carried out on AgNPs sample suspended in MilliQ water, sonicated for 15 min at 20 Hz (Sonic Ruptor 250, Omni Int., Kennesaw, GA, USA) to obtain a homogenous suspension, 1000-fold diluted, and filtered through a 0.22 μm Millipore filter prior to analyses. Zetasizer software was used to analyze the data of AgNPs sample.

### 2.3. Evaluation of antibacterial activity of AgNPs and antibiotics

#### 2.3.1. Bacterial strains

The antibacterial activity of mycosynthesized AgNPs and antibiotics was evaluated against Gram-negative bacterial strains, namely *Escherichia coli* ATCC 25922*, E. coli* ATCC 8739, *Klebsiella pneumoniae* ATCC 700603*, Pseudomonas aeruginosa* ATCC 10145*, Salmonella enterica* PCM 2565, *Salmonella infantis* (strain from Sanitary-Epidemiology Station in Toruń, Poland)*, Agrobacterium tumefaciens* IOR 911, *Pectobacterium carotovorum* PCM 2056, *Pseudomonas syringae* IOR 2188 and *Xanthomonas campestris* IOR 512 and Gram-positive bacteria including *Staphylococcus aureus* ATCC 6538, *S. aureus* ATCC 25923 and *Listeria monocytogenes* PCM 2191.

#### 2.3.2. Minimum inhibitory concentration and minimum biocidal concentration determination for AgNPs and antibiotics

The micro-dilution method was used for estimation of the minimum inhibitory concentration (MIC) of antibiotics and AgNPs according to the Clinical Laboratory Standards Institute standard [Clinical and Laboratory Standards Institute (CLSI), 2012]. Briefly, to estimate the MIC of AgNPs and antibiotics, bacteria were grown in Tryptic Soy Broth (TSB, Becton Dickinson) for 24 h at 37°C and 28°C for human and phytopathogens, respectively. Then, the density of bacterial suspension in sterile distilled water was established at 0.5 McFarland unit (approx. 1.5 × 10^8^ colony forming units per mL; CFU mL^−1^) and ten-fold dilution. Antimicrobials were tested, in triplicate, using sterile 96-well plates (Nest) in the concentration range 0.125–2048 for AgNPs and 0.016–2048 μg mL^−1^ for antibiotics. The TSB medium was used as a diluent for AgNPs and growth medium. The final volume and bacterial concentration in each well were 150 μl and 1.5 × 10^5^ CFU mL^−1^, respectively. Inoculated plates were incubated for 24 h at 37 or 28°C, respectively. The MIC value of AgNPs and antibiotics was defined as the lowest concentration of the antimicrobial agent showing no visible bacterial growth after incubation time. To determine the minimum biocidal concentration (MBC) of AgNPs and antibiotics, samples from all wells without visible bacterial growth were spread onto Trypticase Soy Agar (TSA, Becton Dickinson) in Petri plates and incubated for 24 h at 37 or 28°C, respectively. The MBC values were read as the lowest concentration of antimicrobial agent that inhibited bacterial growth ≥99.9% (Wypij et al., 2021).

#### 2.3.3. Fractional inhibitory concentration index determination

This assay was performed, in triplicate, using sterile 96-well plates (Nest) and TSB medium for bacterial growth. Combined antimicrobial agents (AgNPs and antibiotic) were tested in the concentration range from 1/32 to 2x MIC. The final concentration of bacteria in each well was as for the MIC assay described above. The combined antibacterial effect of AgNPs and antibiotics, as FIC index, was calculated using the formula:


FICindex=MICAgNPs in combination with antibioticMICAgNPs alone+MICAgNPs in combination with antibioticMICantibiotic alone

Values of the FIC index were interpreted as follows: >2.0 denote antagonistic activity, from 0.5 to 2.0 signify an additive effect, while values <0.5 specify a synergistic effect of antimicrobials (Ruden et al., 2009).

### 2.4. Antibiofilm activity of AgNPs

#### 2.4.1. Inhibition of biofilm formation

The ability to biofilm formation by tested bacteria in presence of AgNPs was evaluated, in triplicate, in 96-well flat-bottom plates using crystal violet staining assay (Feoktistova et al., 2016). Each well contained 150 μl of TSB, desired concentration of AgNPs in the range 0.125–512 μg mL^−1^ (2-fold dilutions of AgNPs were maintained from 0.125 to 512 μg mL^−1^), and tested bacteria (final concentration approx. 1.5 × 10^5^ CFU mL^−1^). The plates were incubated for 24 h at 37 or 28°C, respectively. The positive (inoculated TSB without nanoparticles) and negative (sterile TSB) controls as well as background (nanoparticles in TSB) were maintained during the test. After incubation, the suspension was gently removed and wells were washed with sterile distilled water to eliminate planktonic cells. Adherent cells were fixed by drying at room temperature for 1 h and stained with 1% water solution of crystal violet (BTL) for 15 min. Crystal violet was removed and biofilm was washed thrice with sterile distilled water. The aliquots (200 μl) of 99.9% ethanol (Avantor) were added to each well to release absorbed crystal violet from biofilm and absorbance at the wavelength of 595 nm was read using a plate reader (SpectraMax iD3 Multi-Mode Microplate Reader, Molecular Devices, USA). The data were averaged, and the standard deviation was also calculated. Results are presented as percent of biofilm formation in the presence of AgNPs compared to control and calculated using the following formula:


$$\mathrm{Biofilm formation}\;\left[\%\right]=\frac{A_{595}\;\mathrm{after AgNPs treatment}}{A_{595}\mathrm{without AgNPs treatment}}\times 100\%$$

#### 2.4.2. Evaluation of hydrolytic activity of biofilm

Hydrolytic activity of biofilms developed in presence of different concentrations (mentioned above) of AgNPs from *F. culmorum* strain JTW1 were assessed using fluoresceine diacetate (FDA; Sigma-Aldrich) assay (Peeters et al., 2008). Bacterial biofilm was developed in the presence of AgNPs in sterile 96-well flat bottom plates dedicated for fluorescence assays (Thermo Fisher Scientific, USA), as described above. The positive and negative controls as well as background samples were also maintained. Plates were incubated as described previously. After incubation, the suspension was gently removed and wells were washed thrice with 3-(Morpholin-4-yl)propane-1-sulfonic acid (MOPS) buffer (Sigma-Aldrich), pH 7. Subsequently, working solution (0.02%, w/v) of FDA in acetone was prepared, which was then diluted 1:100 (v/v) with MOPS buffer. The aliquots (200 μl) of the FDA solution in MOPS were added to each well and plates were incubated for 4 h at 37°C or 28°C, respectively in the darkness. Fluorescence measurements were performed using plate reader (SpectraMax iD3 Multi-Mode Microplate Reader, Molecular Devices, USA) at an excitation wavelength 494 nm and an emission wavelength 518 nm. Results were shown as percence of released fluorescein compared to the control sample which is directly proportional to the activity of hydrolases produced by developed biofilms. Hydrolytic activity was calculated using the following formula:


$$\mathrm{Hydrolytic activity}\;\left[\%\right]=\frac{\mathrm{Fluorescence after AgNPs treatment}}{\mathrm{Fluorescence without AgNPs treatment}}\times 100\%$$

### 2.5. Antifungal activity

#### 2.5.1. Antifungal assay using agar-well diffusion method

Preliminary screening assay was performed to estimate inhibitory effect of AgNPs against 12 phytopathohenic fungi, namely *Alternaria alternata* IOR 1783 (isolated from kohlrabi), *Botrytis cinerea* IOR 1873 (isolated from tomato), *Colletotrichum acutatum* IOR 2153 (isolated from blueberry), *Fusarium oxysporum* IOR 342 (isolated from pine), *Fusarium solani* IOR 825 (isolated from parsley), *Phoma lingam* IOR 2284 (isolated from rape), *Sclerotinia sclerotiorum* IOR 2242 (isolated from broccoli), and oomycetes, such as *Phytophthora cactorum* IOR 1925 (isolated from strawberry), *Phytophthora cryptogea* IOR 2080 (isolated from Lawson cypress), *Phytophthora megasperma* IOR 404 (isolated from raspberry), *Phytophthora plurivora* IOR 2303 (isolated from *Quercus petraea*) using agar well-diffusion method (Magaldi et al., 2004), with some modifications. Briefly, fungal colonies grown on potato dextrose agar (PDA, Becton Dickinson) in Petri plates for 14 days at 26°C were washed with 10 ml of sterile distilled water to release fungal spores/sclerotia. Their suspensions were collected and filtered through a sterile cotton wool syringe filter to remove mycelia. The concentration of fungal spores/sclerotia were estimated using cell counting chamber (Brand, Germany) and diluted to adjust concentration of 10^6^ spores mL^−1^. One milliliter of such suspension was added into 6 ml of sterile melted PDA and spread on the surface of sterile medium in Petri plates, as a second layer. Subsequently, the wells (Ø =5 mm) were cut in the inoculated plates using sterile cork borer and filled with 50 μl of AgNPs solution at concentration of 3 mg mL^−1^. Then, inoculated plates were incubated for 7 days at 26°C and zones of inhibition of fungal growth around wells were measured in mm.

#### 2.5.2. Determination of minimum inhibitory concentration and minimum fungicidal concentration of AgNPs against fungal spores

Two-fold broth microdilution method [Clinical and Laboratory Standards Institute (CLSI), 2012] adopted for fungal spores, in potato dextrose broth (PDB, A&A Biotechnology), was used to evaluate inhibition of spore germination of the fungal strains, namely *Botrytis cinerea* IOR 1873, *Phoma lingam* IOR 2284 and *Sclerotinia sclerotiorum* IOR 2242 that were selected in agar-well diffusion assay described above. Assay was performed, in triplicate, in sterile 96-well plates (Nest). The AgNPs concentration range was 2–2048 μg mL^−1^. Each well was inoculated with spore/sclerotia suspension (final concentration 10^5^ spores mL^−1^). Negative (sterile broth) and positive (inoculated broth without AgNPs) controls were also maintained. Plates were incubated at 26°C and examined after 7 days for MIC determination. For minimal fungicidal concentration (MFC) the 100 μl from wells without visible fungal growth were spread on the surface of sterile PDA in Petri plates and incubated at 26°C for 7 days.

### 2.6. Statistical analyses

The data were presented as mean ± standard deviation (SD). For results from antibiofilm assays the differences between means were statistically tested by One-way ANOVA followed by Tukey’s test and considered statistically significant if *p* < 0.05. The data were analyzed by Statistica Software (StatSoft Inc., Tulsa, OK, United States).

## 3. Results

### 3.1. Isolation and identification

The ITS sequence of isolate JTW1 showed 100% similarity to corresponding sequence of *Fusarium culmorum* isolate F52 (ITS sequence accession number: MH681149). Strain morphology and growth on potato dextrose agar are shown in Supplementary Figure S1.

### 3.2. Synthesis and characterization of mycosynthesized silver nanoparticles

The color change of the reaction mixture (cell filtrate with silver nitrate) from yellow to dark-brown indicated the formation of AgNPs. The UV–Vis spectroscopy of AgNPs showed the maximum absorption peak at 430 nm (Supplementary Figure S2) which confirmed reduction of Ag^+^ to Ag^0^ and AgNPs formation.

**Figure 1:**
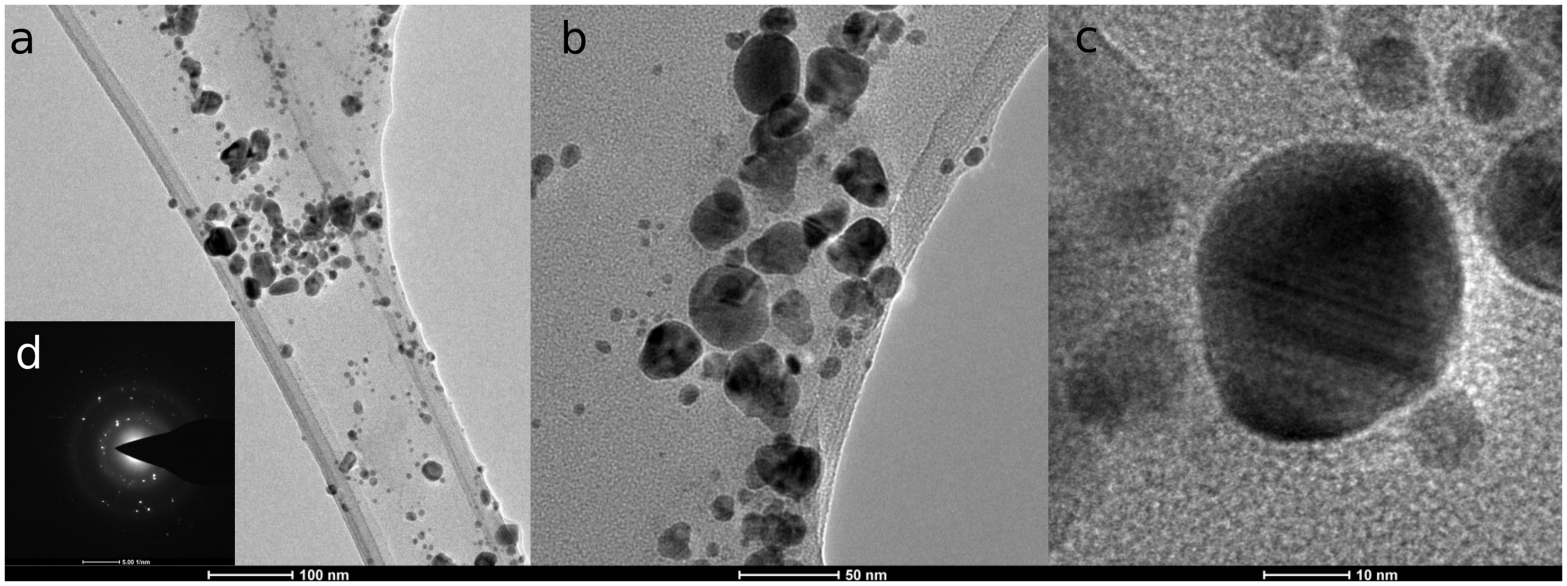
TEM micrographs of silver nanoparticles from *F. culmorum* strain JTW1 **(A–C)** and SAED.

TEM micrographs (Figure 1) revealed that mycosynthesized AgNPs were polydisperse, spherical, with the size range from 3.58 to 58.63 nm. The average size was determined at 15.56 ± 9.22 nm. Energy dispersive X-ray (EDX) spectrum of AgNPs confirmed the presence of silver metal, as shown in Supplementary Figure S3. The elemental analysis data obtained from EDX displayed a 53.00 W% mass of Ag, followed by 44.49 W% of C, and 2.00 W% of O. The EDX pattern showed silver peaks at 3, 22, and 24 keV.

**Figure 2:**
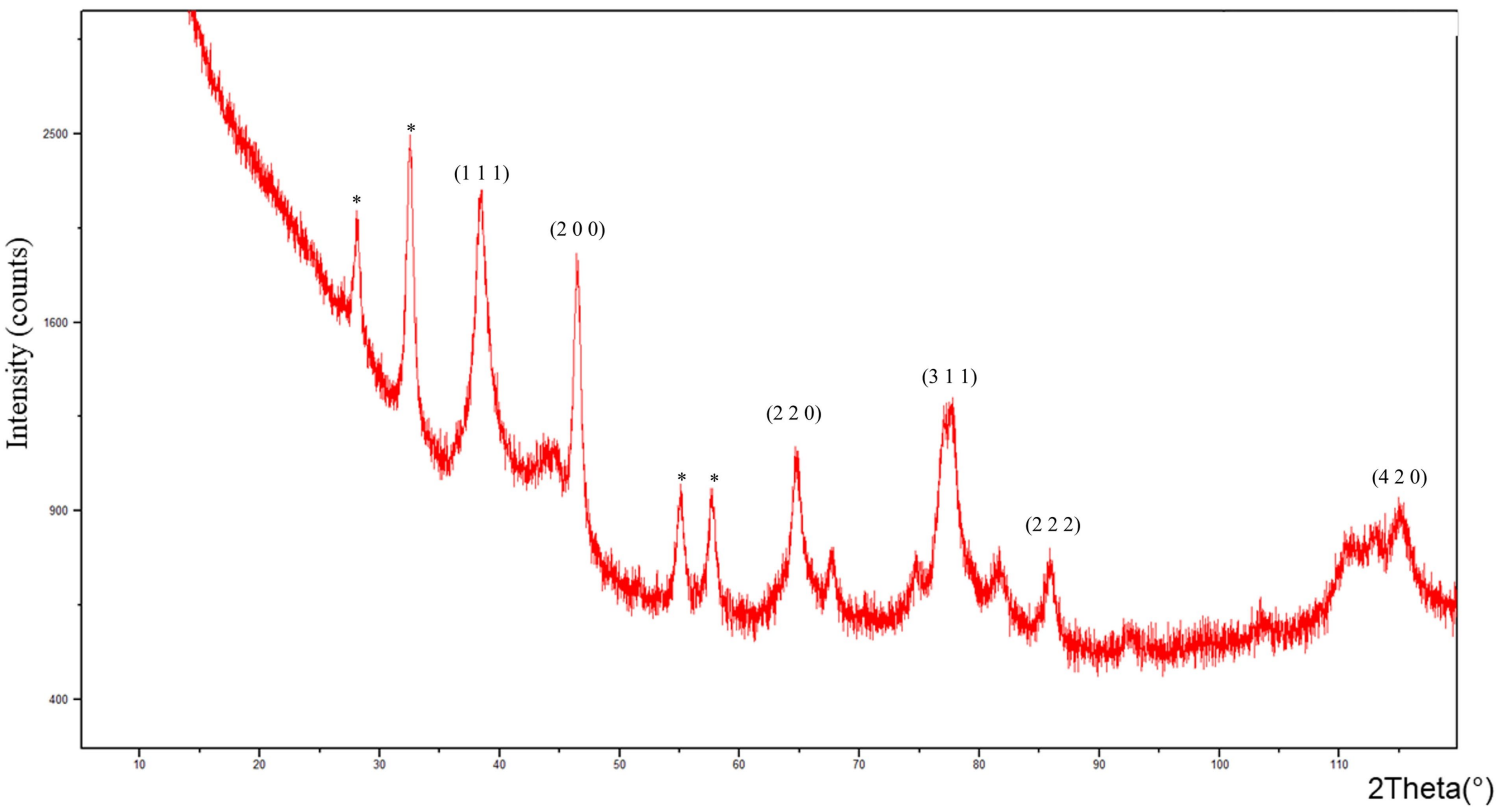
X-ray diffraction (XRD) pattern of AgNPs from *F. culmorum* strain JTW1.

The XRD patterns of AgNPs showed diffraction peaks at: 38.412°, 46.565°, 64.752°, 77.645°, 85.929° and 115.118° corresponding to (1 1 1), (2 0 0), (2 2 0), (3 1 1) (2 2 2), and (4 2 0) planes of the face-centered cubic (fcc) silver crystal, respectively (Figure 2).

**Figure 3:**
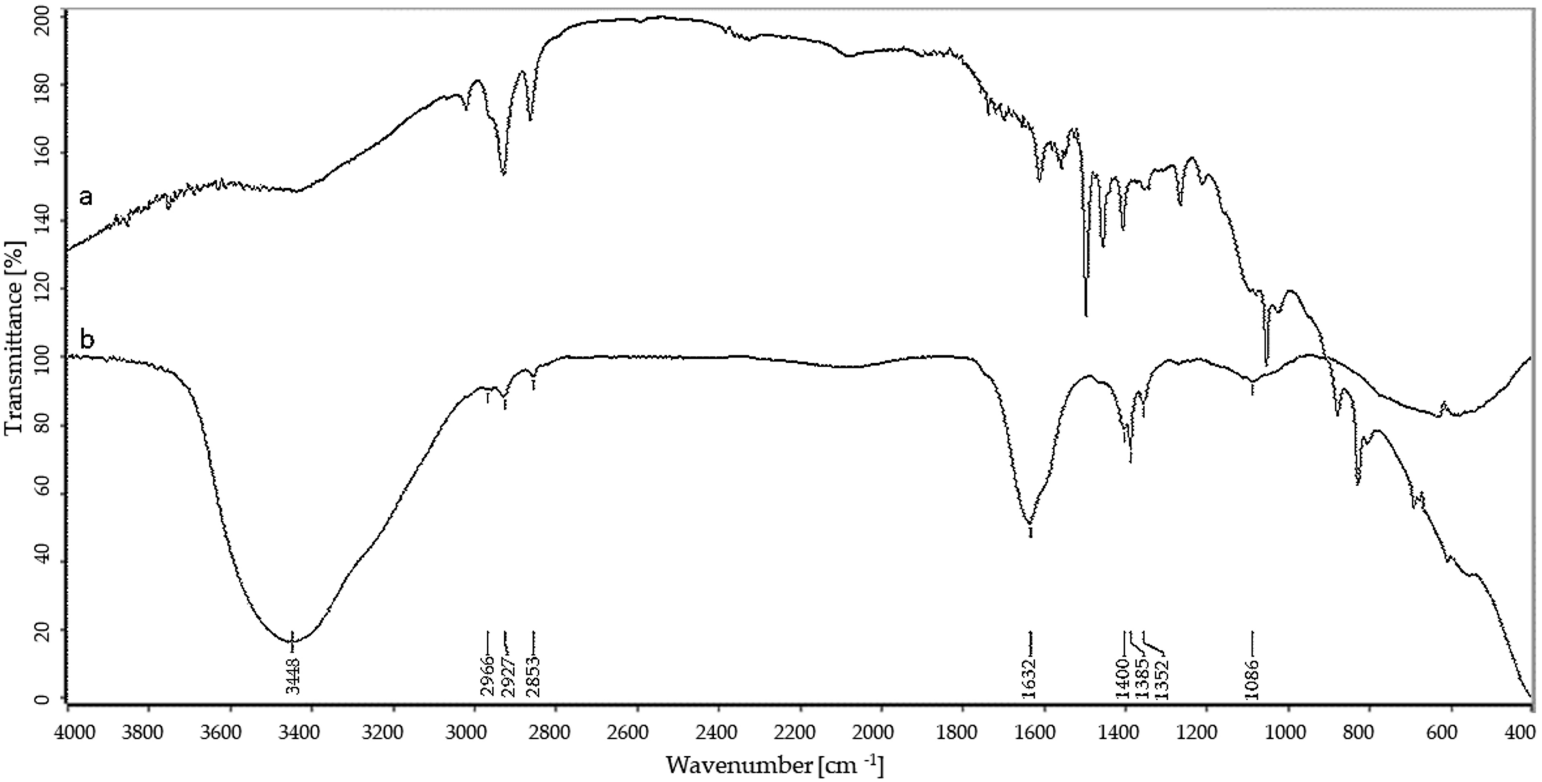
FTIR spectra of control (a) and silver nanoparticles (b).

FTIR analyses of AgNPs, shown in Figure 3, revealed peaks at 3448.75, 2923.34, 2852.38, 1632.52, 1384.60, 1352.28 and 1085.60 cm^−1^.

**Figure 4:**
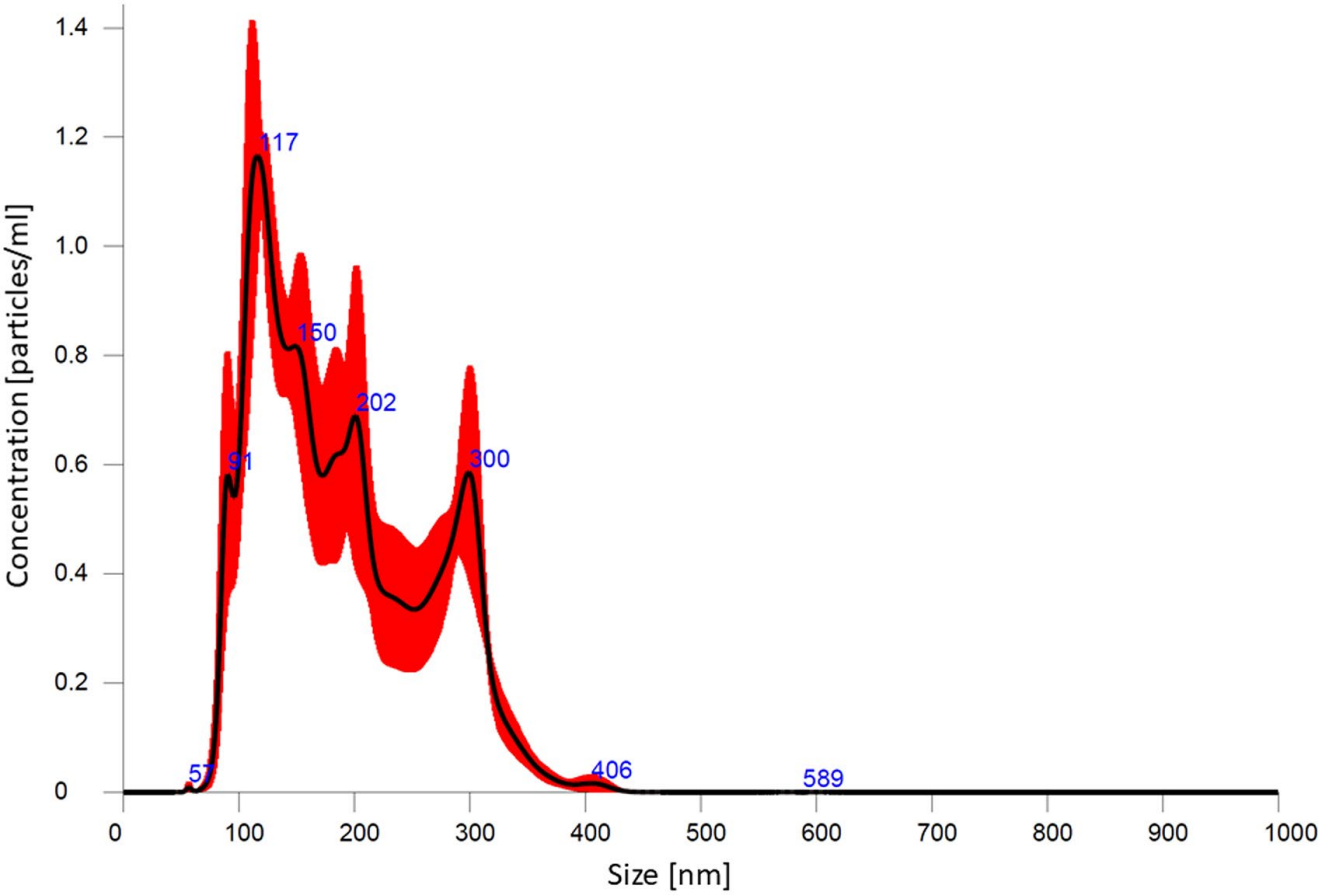
Silver nanoparticle size distribution from nanoparticle tracking analysis.

Particle size distribution provided by NTA is presented in Figure 4. This analysis revealed that average size and concentration of AgNPs were 188.4 nm and 1.46 × 10^12^ particles mL^−1^, respectively. The zeta potential analysis demonstrated that nanoparticles were negatively charged (−38.43 mV), as shown in Supplementary Figure S4. The dynamic light scattering (DLS) presented that the most frequent were AgNPs with a dimension of 169.9 nm (Supplementary Figure S5).

### 3.3. Antimicrobial activity

#### 3.3.1. Minimal inhibitory concentration and minimal biocidal concentration of silver nanoparticles against bacterial strains

The MIC and MBC values of the AgNPs are shown in Table 1. AgNPs synthesized from *F. culmorum* strain JTW1 exhibited strong antibacterial activity against Gram-negative and Gram-positive bacteria. The findings presented here indicate that the activities of AgNPs against different pathogenic microbes were dose-dependent. MIC and MBC of AgNPs against human and food-borne pathogens were in the range of 16–64 and 32–512 μg mL^−1^, respectively.

**Table 1:** Minimal inhibitory (MIC) and minimal biocidal (MBC) concentration values [μg mL^−1^] of AgNPs against bacterial strains.

Tested microorganisms	AgNPs	
	MIC	MBC
*Escherichia coli* ATCC 8739	32	64
*Escherichia coli* ATCC 25922	32	32
*Klebsiella pneumoniae* ATCC 700603	64	128
*Pseudomonas aeruginosa* ATCC 10145	16	32
*Staphylococcus aureus* ATCC 6538	16	64
*Staphylococcus aureus* ATCC 25923	16	128
*Listeria monocytogenes* PCM 2191	64	512
*Salmonella enterica* PCM 2565	32	32
*Salmonella infantis*	64	64
*Agrobacterium tumefaciens* IOR 911	8	256
*Pectobacterium carotovorum* PCM 2056	128	512
*Pseudomonas syringae* IOR 2188	8	128
*Xanthomonas campestris* IOR 512	32	64

The AgNPs from *F. culmorum* strain JTW1 showed the maximum antibacterial activity against phytopathogens as follows: *P. syringae* (MIC = 8 μg mL^−1^ and MBC = 128 μg mL^−1^), *A. tumefaciens* (MIC = 8 μg mL^−1^ and MBC = 256 μg mL^−1^), *X. campestris* (MIC = 32 μg mL^−1^ and MBC = 64 μg mL^−1^) and *P. carotovorum* (MIC = 128 μg mL^−1^ and MBC = 512 μg mL^−1^).

#### 3.3.2. MICs and MBCs of antibiotics against bacteria

The MIC and MBC values of the antibiotics are shown in Table 2. All microorganisms were sensitive to antibiotics in a dose-dependent manner. Minimum inhibitory concentration (MIC) values ranged from 0.016–16, 4–64, 4–128, and 0.064–2048 μg mL^−1^ for tetracycline, streptomycin, kanamycin, and ampicillin, respectively. Overall, Gram-negative bacteria showed lower susceptibility to tested antibiotics than Gram-positive ones. *K. pneumoniae* and *P. aeruginosa* strains were most resistant to tested antibiotics. All of the antibiotics were most efficient (MIC and MBC values in the range 0.016–16 μg mL^−1^) against both *S. aureus* strains. The only exception was tetracycline, for which the MBC was not determined up to the test concentration of 2048 μg mL^−1^.

**Table 2:** MIC and MBC values [μg mL^−1^] of antibiotics: streptomycin, kanamycin, ampicillin, and tetracycline against human pathogenic bacteria.

	Ampicillin		Kanamycin		Streptomycin		Tetracycline	
Tested microorganisms	MIC	MIC	MBC	MBC	MIC	MBC	MIC	MBC
*Escherichia coli* ATCC 8739	4	4	16	16	16	64	0.5	>2048
*Escherichia coli* ATCC 25922	4	4	16	16	16	16	0.25	0.5
*Klebsiella pneumoniae* ATCC 700603	2048	>2048	128	>2048	4	4	16	512
*Pseudomonas aeruginosa* ATCC 10145	512	512	128	128	64	64	8	32
*Staphylococcus aureus* ATCC 6538	0.064	0.064	4	4	4	4	0.016	>2048
*Staphylococcus aureus* ATCC 25923	0.064	0.125	4	4	16	16	0.064	>2048
*Listeria monocytogenes* PCM 2191	0.25	16	4	16	16	128	0.25	64
*Salmonella enterica* PCM 2565	0.25	0.5	8	8	32	2048	0.25	32
*Salmonella infantis*	1	2	16	16	32	1,024	0.5	128

#### 3.3.3. Effects of AgNPs combinations with different antibiotics against human pathogens – FIC index determination

The combined effects of AgNPs from *F. culmorum* strain JTW1 and conventional antibiotics against bacterial strains were demonstrated by using a checkboard method and determination of the FIC index (Table 3). Synergistic effects were found for AgNPs and streptomycin against both *E. coli* strains (FIC = 0.0625), *K. pneumoniae* (FIC = 0.125), and *P. aeruginosa* (FIC = 0.125). MIC values of AgNPs and streptomycin, in combination, were decreased 16-fold against *E. coli,* and 8-fold against *K. pneumoniae* and *P. aeruginosa* when compared to antimicrobials used separately. The combination of AgNPs with ampicillin significantly enhanced the antibacterial activity of antimicrobials against *S. aureus* ATCC 25923 (FIC index = 0.125) and *P. aeruginosa* (FIC index = 0.25). The implemented dose of AgNPs and ampicillin were 8 and 4 times lower, respectively, than MIC values of antimicrobials used alone. Synergistic effects of AgNPs in combination with kanamycin was observed only against *S. aureus* ATCC 6538 (FIC index = 0.25); concentration of AgNPs and antibiotic were reduced four times. In turn, a combination of AgNPs and tetracycline revealed an additive effect against test bacteria (FIC index values were 0.5 or 1).

**Table 3:** FIC index values of AgNPs in combination with antibiotics: ampicillin (AM), kanamycin (K); streptomycin (S), and tetracycline (TE) against bacteria.

Test microorganisms	FIC index			
	AgNPs+AM	AgNPs+K	AgNPs+S	AgNPs+ TE
*Escherichia coli* ATCC 8739	1	1	0.0625	1
*Escherichia coli* ATCC 25922	1	1	0.0652	1
*Klebsiella pneumoniae* ATCC 700603	1	1	0.125	1
*Pseudomonas aeruginosa* ATCC 10145	0.25	1	0.125	1
*Staphylococcus aureus* ATCC 6538	1	0.25	1	1
*Staphylococcus aureus* ATCC 25923	0.125	1	1	1
*Listeria monocytogenes* PCM 2191	1	0.5	0.5	0.5
*Salmonella enterica* PCM 2565	1	0.5	1	0.5
*Salmonella infantis*	1	0.5	1	0.5

FIC values > 2.0 denote antagonistic activity, from 0.5 to 2.0 signify an additive effect, while values < 0.5 specify a synergistic effect of antimicrobials (Ruden et al., 2009).

### 3.4. Antibiofilm formation activity of AgNPs

**Figure 5:**
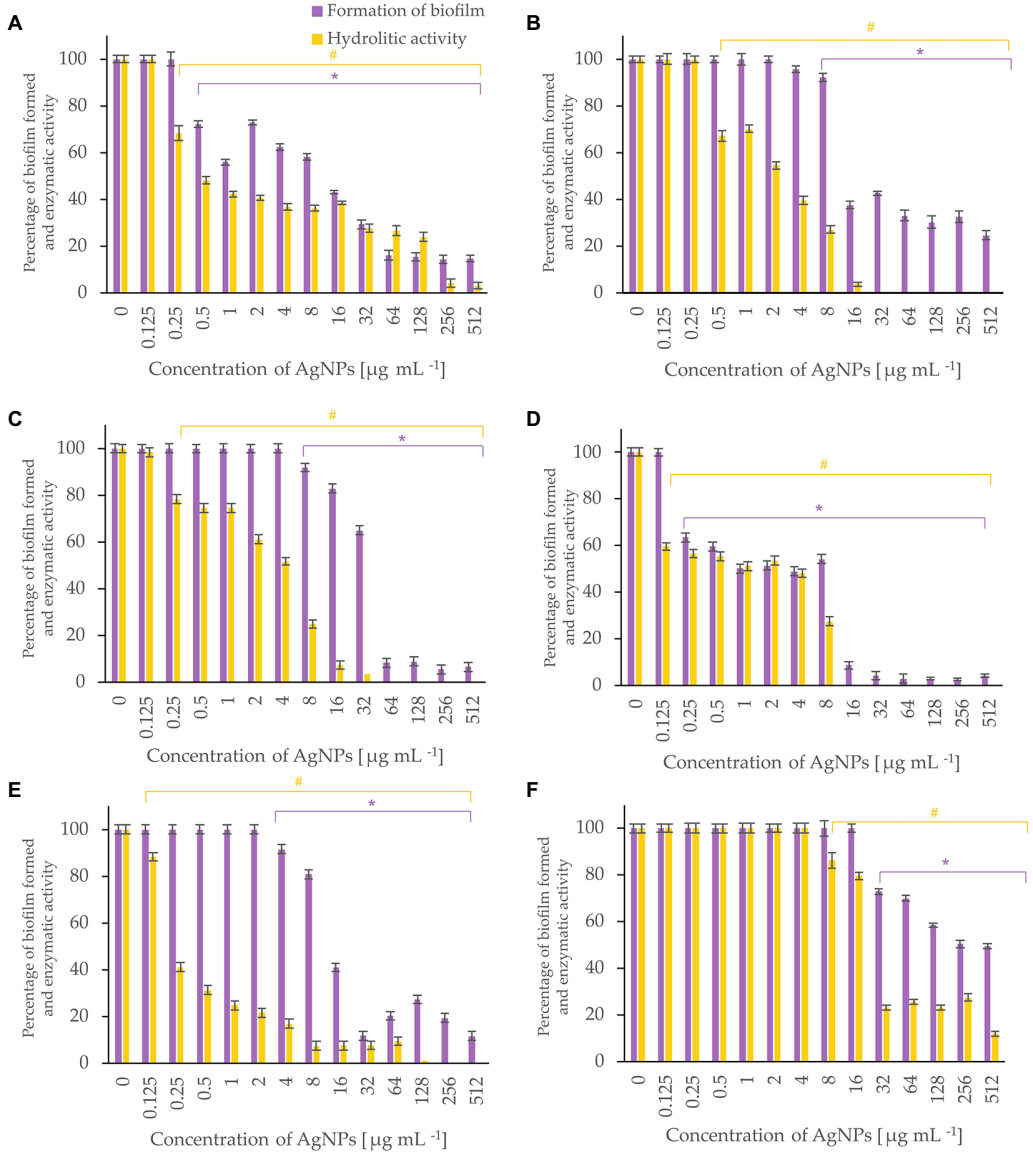
Percent of bacterial biofilm formation and hydrolase activity in biofilms after treatment with various concentrations of AgNPs from *F. culmorum* strain JTW1. *E. coli* ATCC 25922 **(A)**, *E. coli* ATCC 8739 **(B)**, *K. pneumoniae* ATCC 700603 **(C)**, *P. aeruginosa* ATCC 10145 **(D)**, *S. aureus* ATCC 25923 **(E)**, *S. aureus* ATCC 6538 **(F)**. The statistical significance (value of *p* < 0.05) of tested variants (different concentrations of AgNPs) compared with untreated control were indicated by * and # signs for biofilm formation and hydrolytic activity tests, respectively.

**Figure 6:**
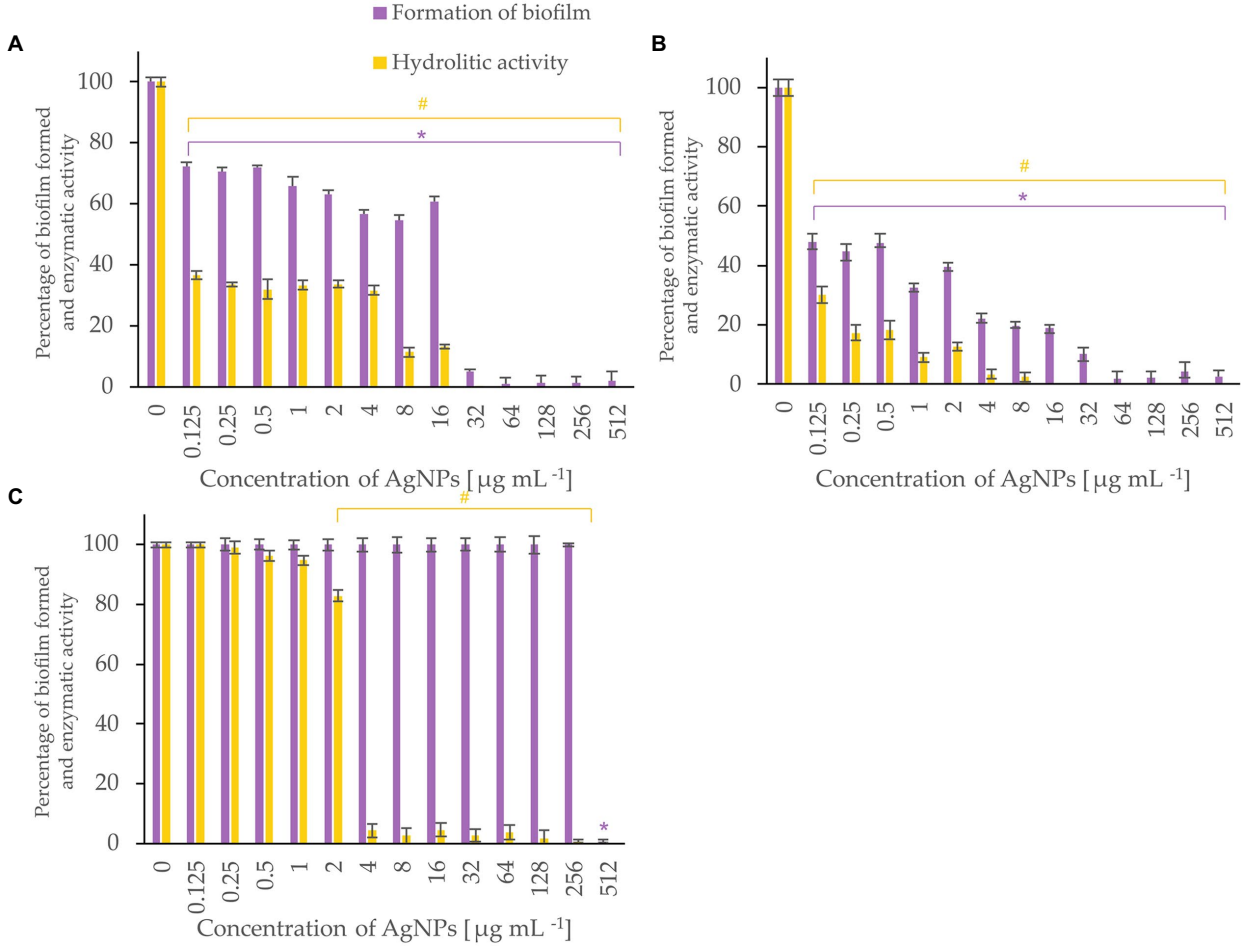
Percent of bacterial biofilm formation and hydrolase activity in biofilms after treatment with various concentrations of AgNPs from *F. culmorum* strain JTW1. *Listeria monocytogenes* **(A)**, *Salmonella enterica* **(B)**, *S. infantis* **(C)**. The statistical significance (value of *p* < 0.05) of tested variants (different concentrations of AgNPs) compared with untreated control were indicated by * and # signs for biofilm formation and hydrolytic activity tests, respectively.

**Figure 7:**
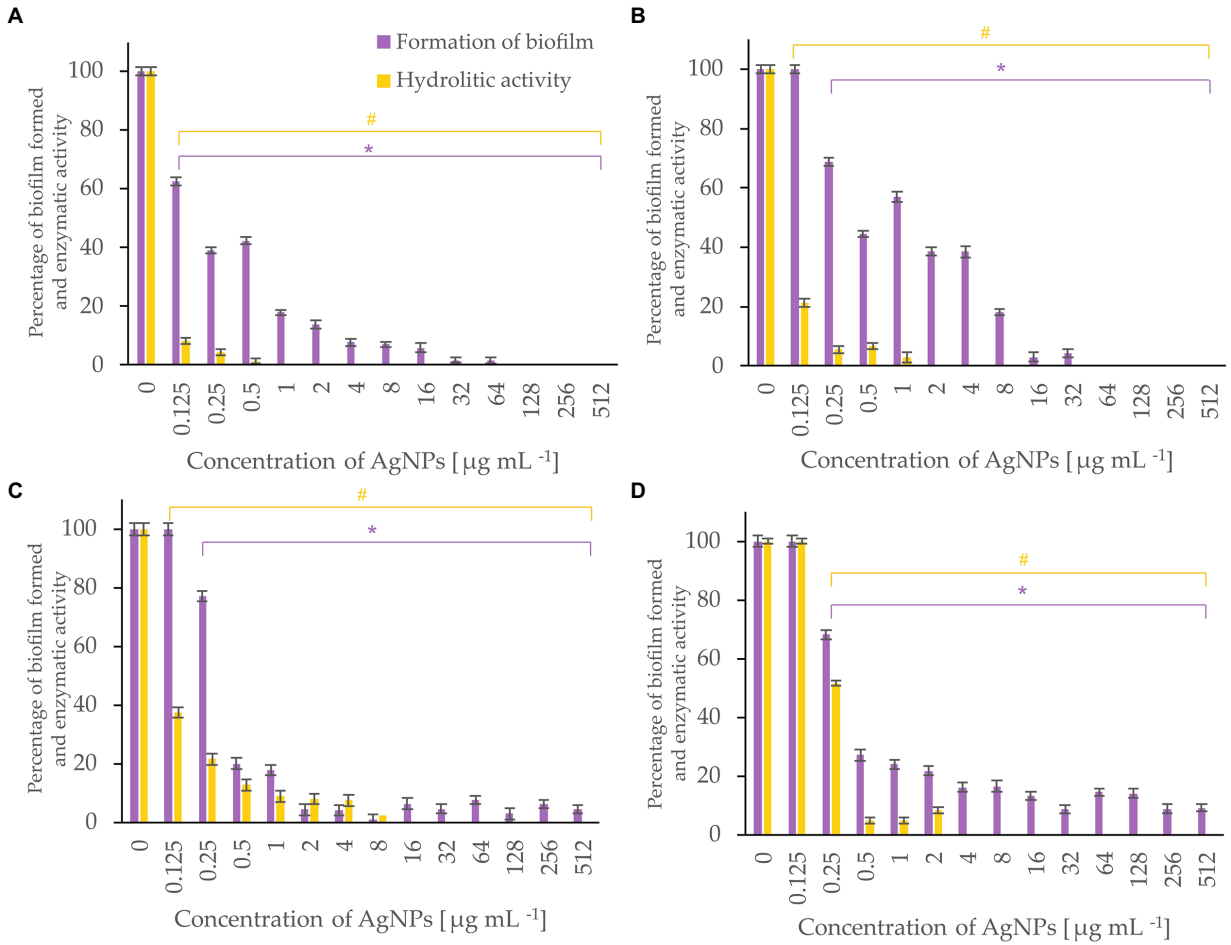
Percent of bacterial biofilm formation and hydrolase activity in biofilms after treatment with various concentrations of AgNPs from *F. culmorum* strain JTW1. *A. tumefaciens* **(A)**, *P. carotovorum* **(B)**, *P. syringae* **(C)**, *X. campestris* **(D)**. The statistical significance (value of *p* < 0.05) of tested variants (different concentrations of AgNPs) compared with untreated control were indicated by * and # signs for biofilm formation and hydrolytic activity tests, respectively.

Bacterial biofilm formation was assessed using crystal violet assay while the hydrolytic activity of the formed biofilms was determined by FDA assay (Figures 5-7). Percentages of biofilm formation and hydrolytic activity of biofilms after AgNPs treatment was estimated compared to control samples (without AgNPs). Metabolic activity and formation of biofilms were inhibited by AgNPs at a concentration range of 0.125–512 μg mL^−1^, in a dose-dependent manner, for all tested microorganisms.

The most effective inhibition of biofilm formation was observed for *Pseudomonas aeruginosa*. In this case, significant inhibition (by 37%) of biofilm formation was found in the presence of 0.25 μg mL^−1^ AgNPs. The AgNPs at a concentration of 0.5 μg mL^−1^ inhibited formation of biofilm by *E. coli* ATCC 25922, by nearly 30%, while at concentration of 64 μg mL^−1^ by >80%. Similarly, AgNPs at concentration of 64 μg mL^−1^ reduced by >60% formation of biofilm by *E. coli* ATCC 8739. In turn, the biofilm formation by *P. aeruginosa* and *K. pneumoniae* was strongly (by 91%) inhibited after treatment with 16 and 64 μg mL^−1^ of AgNPs, respectively. The maximum tested concentration (512 μg mL^−1^) prevented the formation of *S. aureus* ATCC 6538 and *S. aureus* ATCC 25923 biofilms by 50 and 88%, respectively.

AgNPs significantly inhibited the metabolic activity of biofilms formed by strains of *E. coli, K. pneumoniae, P. aeruginosa* and *S. aureus* (Figure 5). Analyses of hydrolases activity in biofilm formed by *P. aeruginosa* in the presence of AgNPs at concentration of 0.25 μg mL^−1^ showed significant decrease in enzyme activity (by 56.6%) while at concentrations ≥16 μg mL^−1^ complete inhibition of activity. The latter is in line with the results of biofilm formation which was strongly prevented. The AgNPs concentrations of 32, 64, and 256 μg mL^−1^ completely inactivated the hydrolytic enzymes of *E. coli* ATCC 8739, *K. pneumoniae,* and *S. aureus* ATCC 25923 biofilms, respectively. The highest tested concentration (512 μg mL^−1^) of AgNPs clearly reduced (by 97.02%) the hydrolytic activity of *E. coli* ATCC 25922 biofilm. The hydrolytic activity of the *S. aureus* ATCC 6538 biofilm remained high until the AgNPs concentration of 16 μg mL^−1^, while higher concentrations caused a decrease in enzyme activity ranging from 23 to 11.7% (32–512 μg mL^−1^).

Metabolic activity of biofilms of *Listeria monocytogenes* and *Salmonella enterica* were notably inhibited with AgNPs at a concentration of 0.125 μg mL^−1^ while the biofilm formation was completely inhibited after treatment of the bacteria with AgNPs at a concentration of 64 μg mL^−1^ or higher (Figure 6). Biofilm of *S. infantis* was extensively formed up to a concentration of 256 μg mL^−1^ of AgNPs and exhibited hydrolytic activity (> 82%) up to AgNPs concentration of 2 μg mL^−1^, while higher AgNPs concentrations resulted in enzyme inactivation (Figure 6).

AgNPs significantly inhibited the enzymatic activity of hydrolases and the formation of biofilm by bacterial plant pathogens (Figure 7). Among them, the most susceptible was *A. tumefaciens* which after exposure to the lowest tested concentration (0.125 μg mL^−1^) of AgNPs caused a decrease in hydrolytic activity to 8% and biofilm formation to 63%. An increase in the dose of AgNPs to 1 μg mL^−1^ completely inhibited hydrolase activity and reduced biofilm formation by 83%. Biofilm formation of *P. syringae, X. campestris,* and *P. carotovorum* was reduced by 80, 73, and 56%, and hydrolytic activity remained below 10% in the presence of 0.5 μg mL^−1^ of AgNPs.

### 3.5. Antifungal activity

Three out of 11 fungal phytopathogens were susceptible to AgNPs from *F. culmorum* strain JTW1 tested using agar well diffusion method. The highest efficiency was observed against *Botrytis cinerea* IOR 1873, followed by *Phoma lingam* IOR 2284 and *Sclerotinia sclerotiorum* IOR 2242. The zones of growth inhibition were found to be 9.54, 4.93, and 3.41 mm, respectively (Table 4; Supplementary Figure S5). AgNPs exhibited strong inhibitory effects against fungal spore germination (Table 4). MIC and MFC values of AgNPs against spore germination of three tested fungal strains were in the range of 32–256 μg mL^−1^. Among them, sclerotia of *Sclerotinia sclerotiorum* displayed the highest susceptibility (MIC and MFC at 32 μg mL^−1^), followed by spores of *Botrytis cinerea* (MIC and MFC at 64 μg mL^−1^) and *Phoma lingam* (MIC and MFC at 256 μg mL^−1^).

**Table 4:** Antifungal activity of AgNPs form *Fusarium culmorum* strain JTW1 against plant pathogens.

Tested strains	Zone of growth inhibition [mm]	MIC [μg mL^−1^]	MFC [μg mL^−1^]
*Botrytis cinerea* IOR 1873	4.93	64	64
*Phoma lingam* IOR 2284	9.54	256	256
*Sclerotinia sclerotiorum* IOR 2242	3.41	32	32

## 4. Discussion

We synthesized AgNPs by mycelial extract of *Fusarium culmorum* isolate JTW1 after treatment with 1 mM aqueous solution of silver nitrate, and visually characterized by the color change, from light-yellow to dark-brown. The color change provides evidence of the formation of AgNPs due to the surface plasmon resonance (SPR) exhibited by the synthesized AgNPs (Smitha et al., 2008). Synthesis of myco-AgNPs was confirmed spectroscopically by observation of a characteristic absorbance peak at 430 nm, which is characteristic of AgNPs. According to the literature, the absorption peaks between 380–450 nm are related to the surface plasmon resonance (SPR) of silver nanoparticles (Desai et al., 2012; Mistry et al., 2021). Our UV–Vis results corroborate with those presented by Huang et al. (2018) and Osorio-Echavarría et al. (2021) who showed that AgNPs synthesized from fungi releveled strong peaks at 430 nm.

Mycosynthesized silver nanoparticles are well known for their bioactivities including antimicrobial activity against both Gram-positive and Gram-negative bacteria (Majeed et al., 2018). The bioactivity of AgNPs is highly dependent on the size, shape, stability, and capping molecules of nanoparticles as well as target cells (e.g., bacterial strain; Li et al., 2012). Therefore, comprehensive characterization of the physical and chemical properties of mycosynthesized AgNPs is important for their potential application.

In the present study, various analytical techniques were used to characterize silver nanoparticles, namely TEM, EDX, XRD, FTIR, DLS, NTA, and Zeta potential measurement. TEM analysis showed small (average size 16 nm) and spherical nanoparticles from *F. culmorum*. Similarly, Bawaskar et al. (2010) reported the synthesis of spherical and small (average size 11 nm) AgNPs by *F. culmorum* extract after challenging with 1 mM AgNO_3_ at ambient temperature. Our results are also in accordance with the findings of Azmath et al. (2016), who reported the fabrication of AgNPs from *Colletotrichum* sp. ALF2-6 with a size ranging from 5 to 60 nm, but with highly variable shapes. However, TEM micrographs showed a clearly smaller size of AgNPs from *F. culmorum* strain JTW1 than NTA and DLS analyses (188.4 and 169.9 nm, respectively) used for further characterization. In the case of the latter analyses the larger size of nanoparticles is due to the presence of capping molecules from fungal extract on the surface of AgNPs (Joshi et al., 2013). Moreover, smaller nanoparticles may not be detected in the DLS technique, as they can be covered by bigger ones (Tomaszewska et al., 2013). In turn, EDX spectrum confirmed elemental composition of AgNPs and the peaks observed at 3, 22, and 24 keV were typical for the absorption of AgNPs due to surface plasmon resonance (Soliman et al., 2022). The X-ray diffraction pattern confirmed the reduction of Ag ^+^ ions and the formation of AgNPs with a face-centered cubic (fcc) structure. However, several unassigned peaks were also recorded in the XRD analysis. It is suggested that these additional peaks arise due to the crystallization of the bioorganic phase on the surface of the AgNPs (Elumalai et al., 2017; Wypij et al., 2022). The presence of biomolecules on the surface of synthesized AgNPs was confirmed by FT-IR measurements which revealed characteristic peaks. The band recorded at 3448.75 cm^−1^ was assigned to the stretching vibrations of primary and secondary amines and indicated the presence of free OH and NH groups. The peaks at 2923.34 and 2852.38 cm^−1^ showed the presence of CH stretching (Ghaseminezhad et al., 2012; Wypij et al., 2021). A band at 1632.52 cm^− 1^ corresponded to the bending vibrations of the amide I and amide II bands of proteins (Elamawi et al., 2018). The bands observed at 1384.60 and 1352.28 cm^−1^ can be assigned to the C–N stretching vibrations of aromatic amines (Jain et al., 2011; Salem, 2022) while the peak at 1085.60 cm^−1^ can be associated with the C–N stretching vibrations of the aliphatic amines (Vigneshwaran et al., 2007). The presence of different functional groups associated with amines, alkanes, and a carboxylic acid, on the surface of AgNPs suggest that various biomolecules from fungal extracts can be involved in nanoparticle formation and stabilization (Ammar and El-Desouky, 2016; Akther et al., 2019). Besides, the biological capping of AgNPs prevent their aggregation and may affect their biological activity, including antimicrobial activity (Hamouda et al., 2019). The high negative value of zeta potential (−38.43 mV) confirmed the repulsion among the particles and indicates their high stability. Elamawi et al. (2018) reported negatively charged (−19.7 mV) and well-dispersed silver nanoparticles synthesized from *Trichoderma longibrachiatum*. However, their zeta potential was much lower suggesting lower stability.

AgNPs synthesized from *F. culmorum* strain JTW1 demonstrated remarkable antimicrobial activity against human pathogens (*Escherichia coli, Klebsiella pneumoniae, Pseudomonas aeruginosa,* and *Staphylococcus aureus*), food-borne pathogens (*Listeria monocytogenes, Salmonella enterica, Salmonella infantis*) and plant pathogens (*Agrobacterium tumefaciens, Pectobacterium carotovorum, Pseudomonas syringae,* and *Xanthomonas campestris*). Interestingly, among human pathogens, the most sensitive to mycosynthesized AgNPs was *P. aeruginosa*, followed by *E. coli, K. pneumoniae,* and food-borne pathogens, including Gram-positive *L. monocytogenes*. It is particularly significant that AgNPs from *F. culmorum* JTW1 inhibited the growth of members of Gram-negative bacteria that are on the list of multidrug-resistant taxa highlighted by the World Health Organization (WHO) (2021).

AgNPs have been found to be effective in the management of plant diseases caused by bacteria, such as *Xanthomonas oryzae, X. phaseoli, Ralstonia solanacearum, Clavibacter michiganensis* and many more (Tariq et al., 2022). In the present study, *P. carotovorum* was found to be the least sensitive to mycosynthesized AgNPs (MIC and MBC values were 128 and 512 μg mL^−1^, respectively) when compared to *P. syringae, X. campestris,* and *A. tumefaciens.*

It is suggested that effective action of silver nanoparticles against bacteria may be through the progressive release of lipopolysaccharides and proteins as well as the further formation of irregularly shaped pits at the outer membrane of Gram-negative bacteria (Amro et al., 2000; Fayaz et al., 2010). Salvioni et al. (2017) demonstrated that antimicrobial activity of negatively charged AgNPs depended on the concentration and was associated with their incorporation into the membrane of Gram-negative bacteria (*E. coli*) and penetration into the cells. In addition, intracellular leakage and coagulation of nanoparticles on the bacterial surface were also observed. In turn, treatment of Gram-positive *S. aureus* with AgNPs resulted in the appearance of blisters on the surface of cells and cell lysis (Salvioni et al., 2017). Other studies indicated that AgNPs or released Ag^+^ ions after penetration into a cell may generate free radicals (Qing et al., 2018; Tian et al., 2018) and interact with biomolecules and intracellular structures (Li et al., 2010; Adeyemi et al., 2020; Gul et al., 2021). Yuan et al. (2017) reported that AgNPs caused lower lactate dehydrogenase activity (LDH) and decreased levels of adenosine triphosphate (ATP) in *P. aeruginosa* and *S. aureus*. Moreover, ROS generated after AgNPs treatment led to inhibition of metabolic activity and the growth of bacterial cells. It is claimed that due to the different mechanisms of AgNPs action, which affect various aspects of bacterial growth and metabolism, the development of microbial resistance to nanoparticles is limited (Wang L. et al., 2017).

In the present study, tested antibiotics demonstrated antibacterial activity against Gram-negative and Gram-positive bacteria which was increased in combination with AgNPs from *F. culmorum* strain JTW1. The enhanced antimicrobial activity against *E. coli*, *K. pneumoniae,* and *P. aeruginosa* was observed for a combination of AgNPs and streptomycin and against *P. aeruginosa* with ampicillin. In the case of *S. aureus,* increased activity of ampicillin and kanamycin was observed when combined with AgNPs. The increased antimicrobial effect may be due to the binding of antibiotics to AgNPs. The molecules of antibiotics react easily with nanoparticles due to many active groups such as hydroxyl and amido groups (Wypij et al., 2022). Hence, AgNPs may be covalently bound to streptomycin due to the presence of four-terminal –NH_2_ groups in antibiotic structure. In addition, this association might be enhanced by the electrostatic interactions between negatively charged AgNPs and positively charged streptomycin molecules (Wang C. et al., 2017). Moreover, biologically synthesized nanoparticles showed more stable adsorption of ampicillin when compared to chemically synthesized ones due to the presence of an organic layer on their surface, which acts as a stabilizer (Rogowska et al., 2017). It is claimed that AgNPs in combination with antibiotics can promote cell wall disruption and damage, and facilitate the transport of antibiotics into the cell by increasing membrane permeability. Other mechanisms of AgNPs action include inhibition of enzymes responsible for the hydrolysis of antibiotics due to the disruption of their structure and release of Ag^+^ ions (Panáček et al., 2016). Therefore, it is assumed that the combination of AgNPs and antibiotics enhanced their inhibitory activity against pathogenic bacteria. This allows the use of lower doses of antibacterial agents and reduces the risk of resistance to antimicrobial agents (Hemeg, 2017).

AgNPs are reported to hinder the biofilm formation in many bacteria (Markowska et al., 2013; Ansari et al., 2021; Hetta et al., 2021). Crystal violet staining and fluorescein diacetate assay was used for the visualization of adherent biofilm bacteria and measurement of biofilm activity, respectively. Interestingly, AgNPs from *Fusarium culmorum* strain JTW1, at low concentration (0.25 μg mL^−1^), efficiently inhibited the formation and hydrolytic activity of *P. aeruginosa* biofilm. Similar results were reported by Akther et al. (2020) who studied the efficiency of AgNPs synthesized from *Rhizopus arrhizus* BRS-07 in reducing biofilm formation by *P. aeruginosa* and found that the formation of this structure was inhibited by 7–93% in a dose-dependent manner (5–25 μg mL^−1^). Furthermore, AgNPs synthesized from *F. culmorum* strain JTW1 at a higher concentration of 64 μg mL^−1^ also efficiently (by 67–96%) reduced the formation of biofilms in *E. coli* strains, *P. aeruginosa,* and *K. pneumoniae.* These nanoparticles were found to be more active against biofilm than those reported by Ramachandran and Sangeetha (2017) which completely inhibited the formation of *Escherichia coli* (ETEC12), *Klebsiella pneumoniae* (SKP7) and *Pseudomonas aeruginosa* (ETPS11) biofilms after treatment with AgNPs at a concentration of 100 μg mL^−1^. Ansari et al. (2014) found the highest antibiofilm efficiency of commercially available water-soluble AgNPs (5–10 nm) against *Escherichia coli* and *Klebsiella* spp. at the concentration of 50 μg mL^−1^. The inhibition of biofilm formation by AgNPs is a consequence of bacterial growth arrest, prevention of exopolysaccharide formation (Ansari et al., 2014), and disruption of bacterial quorum sensing signaling (Akther et al., 2020). In our study, although AgNPs at the lower concentrations had no impact on biofilm formation by *E. coli* ATCC 8739, *K. pneumoniae,* and *S. aureus* ATCC 25923, they significantly decreased the activity of hydrolases.

Food-borne pathogens, such as *Listeria monocytogenes* and *Salmonella enterica* are known to form biofilms, which ultimately leads to serious problems in the food industry (Harrell et al., 2021; Muthulakshmi et al., 2022). Our findings confirmed that both the aforementioned bacteria showed sensitivity to mycofabricated AgNPs. However, the development of biofilm in *L. monocytogenes* was more prevented than *S. infantis.* Our results are in line with those reported by Chlumsky et al. (2020) who showed higher activity of AgNPs in reducing biofilm formation and a decrease in metabolic activity against *L. monocytogenes, S. aureus* than *S. infantis* S59. The *in vitro* studies on the AgNPs from *Terminalia catappa* leaf extract against *L. monocytogenes* revealed the inhibition of biofilm formation and reduction in the virulence factors such as protease production, at concentrations of 50 and 100 μg mL^−1^ (Muthulakshmi et al., 2022). Similarly, AgNPs from *F. culmorum* strain JTW1 remarkably prevented biofilm formation ability in bacterial plant pathogens, especially in *A. tumefaciens* and *P. carotovorum.* Moreover, a significant reduction in the hydrolase enzymatic activity of these bacteria was recorded. Recently, Olfati et al. (2021) also reported the inhibitory effect of AgNPs synthesized from *Calendula officinalis* extract against the biofilm formation of *P. carotovorum*. Consequently, the obtained results clearly reveal that mycosynthesized AgNPs not only effectively inhibited the activity of enzymes but also the growth of the bacteria. Therefore, it can be hypothesized that the ability to inhibit the hydrolytic enzyme activity of bacteria may occur due to an interaction of AgNPs with proteins, causing an alteration in structure and interfering with enzymatic functions (Wigginton et al., 2010).

Silver nanoparticles from *F. culmorum* strain JTW1 showed strong inhibitory activity against the growth and spore germination of *Botrytis cinerea, Sclerotinia sclerotiorum,* and *Phoma lingam,* but not against remaining fungal strains. Similarly, antifungal activity of AgNPs obtained from strains of the genus *Trichoderma* to plant pathogens was shown in other studies reported by Guilger et al. (2017) and Tomah et al. (2020). For instance, AgNPs synthesized from *Trichoderma* sp. at a concentration of 200 μg mL^−1^ reduced the growth of mycelia of *Sclerotinia sclerotiorum*, formation of new sclerotia and their germination (Tomah et al., 2020). The authors further reported the changes in fungal cells by induction of pore formation in the cell wall and further accumulation of AgNPs inside the fungal cells (Tomah et al., 2020). Similarly, Guilger et al. (2017) found that AgNPs at a concentration of 0.31 × 10^12^ NPs/mL inhibited mycelial growth and the formation of new sclerotia of *Sclerotinia sclerotiorum*. However, in our study, inhibition of sclerotia germination was achieved at a much lower concentration of myco-AgNPs (32 μg mL^−1^) showing their higher effectiveness in elimination of *S. sclerotiorum*. In another study, Malandrakis et al. (2019) tested the sensitivity of a number of fungal pathogens to commercially available silver nanoparticles (size <100 nm) and reported the highest susceptibility of *Botrytis cinerea,* followed by *Verticillium dahliae, Colletotrichum gloeosporioides, Monilia fructicola, Alternaria alternata, Fusarium oxysporum* f. sp. *Radicis-Lycopersici* and *Fusarium solani* (the latter was unaffected in tested concentration range). The higher inhibitory effect of AgNPs was observed against spores germination (EC_50_ values in the range 5.08–390.20 μg mL^−1^) than mycelial growth (EC_50_ value were > 306 μg mL^−1^) of tested pathogens (Malandrakis et al., 2019). Our findings provide evidence of the differential response of fungal pathogens to AgNPs. The highest inhibition of mycelial growth was recorded in *P. lingam* (9.54 mm), followed by *B. cinerea* (4.93 mm) and *S. sclerotiorum* (3.41 mm) whereas, in the case of spore germination, maximum inhibition was found in *S. sclerotiorum* (32 μg mL^−1^), followed by *B. cinerea* (64 μg mL^−1^) and *P. lingam* (256 μg mL^−1^). Nevertheless, our findings of antimicrobial potential of AgNPs against a wide range of fungal crop pathogens indicate their potential for use in agricultural crop management to protect against diseases such as gray or white mold, blackleg, canker, and dry rot.

## 5. Conclusion

In this study, AgNPs were effectively synthesized from *Fusarium culmorum* strain JTW1. They were comprehensively characterized using UV–vis, TEM, XRD, NTA, Zeta potential measurements, and FTIR which revealed small size, spherical shape, stability, crystalline nature of AgNPs, and that mycosynthesized AgNPs were capped with biomolecules. Moreover, AgNPs were found to have strong antimicrobial potential against bacterial pathogens of humans and plants. AgNPs also enhanced antibacterial activity of streptomycin against Gram-negative bacteria, namely *E. coli* strains ATCC 8739 and ATCC 25922, *K. pneumoniae* ATCC 700603, *P. aeruginosa* ATCC 10145, ampicillin against *P. aeruginosa*, and ampicillin and kanamycin against Gram-positive *S. aureus* ATCC 25923 and *S. aureus* ATCC 6538, respectively. Furthermore, AgNPs significantly reduced bacterial biofilm formation and inhibited activity of hydrolytic enzymes synthesized by biofilm cells. In addition, AgNPs showed great potential to inhibit fungal spore germination, which is crucial in the fungal spread in the environment, and growth of fungal mycelia. Therefore, these mycosynthesized silver nanoparticles in view of their unique properties, have a high potential as an antimicrobial agent in medicine and promising solution to control or reduce pathogens in agriculture and the food industry.

## Data availability statement

The original contributions presented in the study are included in the article/Supplementary material, further inquiries can be directed to the corresponding authors.

## Author contributions

PG and MR conceived research and edited and reviewed it. PG and JT-W designed research. JT-W and MW conducted experiments and acquired the funds. JT-W analyzed data and wrote the manuscript. All authors have read and agreed to the published version of the manuscript.

## Funding

This research was funded by Excellence Initiative – Research University, Nicolaus Copernicus University in Torun, Grant4NCUStudents (38/2021/Grants4NCUStudents and 02/2021/Grants4NCUStudents). The ACP was funded from EF-IDUB of Nicolaus Copernicus University.

## Conflict of interest

The authors declare that the research was conducted in the absence of any commercial or financial relationships that could be construed as a potential conflict of interest.

## Publisher’s note

All claims expressed in this article are solely those of the authors and do not necessarily represent those of their affiliated organizations, or those of the publisher, the editors and the reviewers. Any product that may be evaluated in this article, or claim that may be made by its manufacturer, is not guaranteed or endorsed by the publisher.

## References

[ref1] Adeyemi O. S. Shittu E. O. Akpor O. B. Rotimi D. Batiha G. E. S. (2020). Silver nanoparticles restrict microbial growth by promoting oxidative stress and DNA damage. EXCLI J. 19, 492–500. doi: 10.17179/excli2020-1244, PMID: 32398973 PMC7214780

[ref2] Akther T. Khan M. S. Hemalatha S. (2020). Biosynthesis of silver nanoparticles via fungal cell filtrate and their anti-quorum sensing against *Pseudomonas aeruginosa*. J. Environ. Chem. Eng. 8:104365. doi: 10.1016/j.jece.2020.104365

[ref3] Akther T. Mathipi V. Kumar N. S. Davoodbasha M. Srinivasan H. (2019). Fungal-mediated synthesis of pharmaceutically active silver nanoparticles and anticancer property against A549 cells through apoptosis. Environ. Sci. Pollut. Res. 26, 13649–13657. doi: 10.1007/s11356-019-04718-w, PMID: 30919178

[ref4] Ali K. Ahmed B. Dwivedi S. Saquib Q. Al-Khedhairy A. A. Musarrat J. (2015). Microwave accelerated green synthesis of stable silver nanoparticles with Eucalyptus globulus leaf extract and their antibacterial and antibiofilm activity on clinical isolates. PLoS One 10:e0131178. doi: 10.1371/journal.pone.0131178, PMID: 26132199 PMC4489395

[ref5] Allocati N. Masulli M. Alexeyev M. F. Di Ilio C. (2013). *Escherichia coli* in Europe: an overview. Int. J. Environ. Res. Public Health 10, 6235–6254. doi: 10.3390/ijerph10126235, PMID: 24287850 PMC3881111

[ref6] Al-Rajhi A. M. Salem S. S. Alharbi A. A. Abdelghany T. M. (2022). Ecofriendly synthesis of silver nanoparticles using Kei-apple (*Dovyalis caffra*) fruit and their efficacy against cancer cells and clinical pathogenic microorganisms. Arab. J. Chem. 15:103927. doi: 10.1016/j.arabjc.2022.103927

[ref7] Ammar H. A. M. El-Desouky T. A. (2016). Green synthesis of nanosilver particles by *Aspergillus terreus* HA 1N and *Penicillium expansum* HA 2N and its antifungal activity against mycotoxigenic fungi. J. Appl. Microbiol. 121, 89–100. doi: 10.1111/jam.13140, PMID: 27002915

[ref8] Amro N. A. Kotra L. P. Wadu-Mesthrige K. Bulychev A. Mobashery S. Liu G. Y. (2000). High-resolution atomic force microscopy studies of the *Escherichia coli* outer membrane: structural basis for permeability. Langmuir 16, 2789–2796. doi: 10.1021/la991013x

[ref9] Amselem J. Cuomo C. A. van Kan J. A. Viaud M. Benito E. P. Couloux A.. (2011). Genomic analysis of the necrotrophic fungal pathogens *Sclerotinia sclerotiorum and Botrytis cinerea*. PLoS Genet. 7:e1002230. doi: 10.1371/journal.pgen.1002230, PMID: 21876677 PMC3158057

[ref10] Ansari M. A. Asiri S. M. M. Alzohairy M. A. Alomary M. N. Almatroudi A. Khan F. A. (2021). Biofabricated fatty acids-capped silver nanoparticles as potential antibacterial, antifungal, antibiofilm and anticancer agents. Pharmaceuticals 14:139. doi: 10.3390/ph14020139, PMID: 33572296 PMC7915658

[ref11] Ansari M. A. Khan H. M. Khan A. A. Cameotra S. S. Pal R. (2014). Antibiofilm efficacy of silver nanoparticles against biofilm of extended spectrum β-lactamase isolates of *Escherichia coli* and *Klebsiella pneumoniae*. Appl. Nanosci. 4, 859–868. doi: 10.1007/s13204-013-0266-1

[ref13] Azmath P. Baker S. Rakshith D. Satish S. (2016). Mycosynthesis of silver nanoparticles bearing antibacterial activity. Saudi Pharm. J. 24, 140–146. doi: 10.1016/j.jsps.2015.01.008, PMID: 27013906 PMC4792906

[ref14] Bawaskar M. Gaikwad S. Ingle A. Rathod D. Gade A. Duran N.. (2010). A new report on mycosynthesis of silver nanoparticles by *Fusarium culmorum*. Curr. Nanosci. 6, 376–380. doi: 10.2174/157341310791658919

[ref16] Butsenko L. Pasichnyk L. Kolomiiets Y. Kalinichenko A. (2020). The effect of pesticides on the tomato bacterial speck disease pathogen *Pseudomonas syringae* pv. *tomato*. Appl. Sci. 10:3263. doi: 10.3390/app10093263

[ref17] Caniça M. Manageiro V. Abriouel H. Moran-Gilad J. Franz C. M. (2019). Antibiotic resistance in foodborne bacteria. Trends Food Sci. Technol. 84, 41–44. doi: 10.1128/AEM.00973-07

[ref18] Charkowski A. O. (2018). The changing face of bacterial soft-rot diseases. Annu. Rev. Phytopathol. 56, 269–288. doi: 10.1146/annurev-phyto-080417-045906, PMID: 29958075

[ref19] Charkowski A. Sharma K. Parker M. L. Secor G. A. Elphinstone J. (2020). “Bacterial diseases of potato,” in The Potato Crop: Its Agricultural, Nutritional and Social Contribution to Humankind. eds. Campos H. Ortiz O. (Cham: Springer), 351–388.

[ref20] Chlebicz A. Śliżewska K. (2018). Campylobacteriosis, salmonellosis, yersiniosis, and listeriosis as zoonotic foodborne diseases: a review. Int. J. Environ. Res. Public Health 15:863. doi: 10.3390/ijerph15050863, PMID: 29701663 PMC5981902

[ref21] Chlumsky O. Purkrtova S. Michova H. Svarcova V. Slepicka P. Fajstavr D.. (2020). The effect of gold and silver nanoparticles, chitosan and their combinations on bacterial biofilms of food-borne pathogens. Biofouling 36, 222–233. doi: 10.1080/08927014.2020.1751132, PMID: 32316774

[ref22] Clinical and Laboratory Standards Institute (CLSI) (2012). Methods for Dilution Antimicrobial Susceptibility Tests for Bacteria that Grow Aerobically; Approved Standard 9th. Document M07-A9, CLSI Wayne, USA.

[ref23] Dadgostar P. (2019). Antimicrobial resistance: implications and costs. Infect. Drug Resist. 12, 3903–3910. doi: 10.2147/IDR.S234610, PMID: 31908502 PMC6929930

[ref24] Dean R. Van Kan J. A. Pretorius Z. A. Hammond-Kosack K. E. Di Pietro A. Spanu P. D.. (2012). The top 10 fungal pathogens in molecular plant pathology. Physiol. Mol. Plant Pathol. 13, 414–430. doi: 10.1111/j.1364-3703.2011.00783.x, PMID: 22471698 PMC6638784

[ref25] Desai R. Mankad V. Gupta S. K. Jha P. K. (2012). Size distribution of silver nanoparticles: UV-visible spectroscopic assessment. Nanosci. Nanotechnol. Lett. 4, 30–34. doi: 10.1166/nnl.2012.1278

[ref26] Elamawi R. M. Al-Harbi R. E. Hendi A. A. (2018). Biosynthesis and characterization of silver nanoparticles using *Trichoderma longibrachiatum* and their effect on phytopathogenic fungi. Egypt. J. Biol. Pest Control 28, 1–11. doi: 10.1186/s41938-018-0028-1

[ref27] Elumalai D. Hemavathi M. Deepaa C. V. Kaleena P. K. (2017). Evaluation of phytosynthesised silver nanoparticles from leaf extracts of *Leucas aspera* and *Hyptis suaveolens* and their larvicidal activity against malaria, dengue and filariasis vectors. Parasite Epidemiol. Control 2, 15–26. doi: 10.1016/j.parepi.2017.09.001 29774292 PMC5952679

[ref28] Fayaz A. M. Balaji K. Girilal M. Yadav R. Kalaichelvan P. T. Venketesan R. (2010). Biogenic synthesis of silver nanoparticles and their synergistic effect with antibiotics: a study against gram-positive and gram-negative bacteria. Nanomed. Nanotechnol. Biol. Med. 6, 103–109. doi: 10.1016/j.nano.2009.04.006, PMID: 19447203

[ref29] Feoktistova M. Geserick P. Leverkus M. (2016). Crystal violet assay for determining viability of cultured cells. Cold Spring Harb. Protoc. 2016:pdb.prot087379. doi: 10.1101/pdb.prot087379 27037069

[ref30] Food and Agriculture Organization of the United Nations (FAO). (2017). The Future of Food and Agriculture – Trends and Challenges. Available at: https://www.fao.org/3/i6583e/i6583e.pdf (Accessed May 5, 2022).

[ref31] Fung F. Wang H. S. Menon S. (2018). Food safety in the 21st century. Biom. J. 41, 88–95. doi: 10.1016/j.bj.2018.03.003, PMID: 29866604 PMC6138766

[ref32] Gabal E. Amal-Asran M. Mohamed M. A. Abd-Elsalam K. A. (2019). “*Botrytis* gray Mold Nano-or biocontrol: present status and future prospects” in Nanobiotechnology Applications in Plant Protection: Volume 2. eds. Abd-Elsalam K. Prasad R. (Cham: Springer), 85–118.

[ref33] Gandhi M. Chikindas M. L. (2007). *Listeria*: a foodborne pathogen that knows how to survive. Int. J. Food Microbiol. 113, 1–15. doi: 10.1016/j.ijfoodmicro.2006.07.008, PMID: 17010463

[ref34] Ghaseminezhad S. M. Hamedi S. Shojaosadati S. A. (2012). Green synthesis of silver nanoparticles by a novel method: comparative study of their properties. Carbohydr. Polym. 89, 467–472. doi: 10.1016/j.carbpol.2012.03.030, PMID: 24750745

[ref35] Guilger M. Pasquoto-Stigliani T. Bilesky-Jose N. Grillo R. Abhilash P. C. Fraceto L. F.. (2017). Biogenic silver nanoparticles based on *Trichoderma harzianum*: synthesis, characterization, toxicity evaluation and biological activity. Sci. Rep. 7, 1–13. doi: 10.1038/srep44421, PMID: 28300141 PMC5353535

[ref36] Gul A. Shaheen A. Ahmad I. Khattak B. Ahmad M. Ullah R.. (2021). Green synthesis, characterization, enzyme inhibition, antimicrobial potential, and cytotoxic activity of plant mediated silver nanoparticle using *Ricinus communis* leaf and root extracts. Biomol. Ther. 11:206. doi: 10.3390/biom11020206, PMID: 33540690 PMC7913007

[ref37] Hall-Stoodley L. Stoodley P. (2005). Biofilm formation and dispersal and the transmission of human pathogens. Trends Microbiol. 13, 7–10. doi: 10.1016/j.tim.2004.11.004 15639625

[ref38] Hamouda R. A. Yousuf W. E. Abdeen E. E. Mohamed A. (2019). Biological and chemical synthesis of silver nanoparticles: characterization, MIC and antibacterial activity against pathogenic bacteria. J. Chem. Pharm. Res. 11, 1–12.

[ref39] Hampf A. C. Nendel C. Strey S. Strey R. (2021). Biotic yield losses in the southern Amazon, Brazil: making use of smartphone-assisted plant disease diagnosis data. Front. Plant Sci. 12:548. doi: 10.3389/fpls.2021.621168, PMID: 33936124 PMC8083370

[ref01] Harrell J. E. Hahn M. M. D’Souza S. J. Vasicek E. M. Sandala J. L. Gunn J. S.. (2021). Salmonella biofilm formation, chronic infection, and immunity within the intestine and hepatobiliary tract. Front. Cell. Infect. Microbiol. 10:624622. doi: 10.3389/fcimb.2020.624622 33604308 PMC7885405

[ref40] Hashempour-Baltork F. Hosseini H. Shojaee-Aliabadi S. Torbati M. Alizadeh A. M. Alizadeh M. (2019). Drug resistance and the prevention strategies in food borne bacteria: an update review. Adv. Pharm. Bull. 9, 335–347. doi: 10.15171/apb.2019.041, PMID: 31592430 PMC6773942

[ref41] Hemeg H. A. (2017). Nanomaterials for alternative antibacterial therapy. Int. J. Nanomed. 12, 8211–8225. doi: 10.2147/IJN.S132163, PMID: 29184409 PMC5689025

[ref42] Hetta H. F. Al-Kadmy I. Khazaal S. S. Abbas S. Suhail A. El-Mokhtar M. A.. (2021). Antibiofilm and antivirulence potential of silver nanoparticles against multidrug-resistant *Acinetobacter baumannii*. Sci. Rep. 11, 10751–10711. doi: 10.1038/s41598-021-90208-4, PMID: 34031472 PMC8144575

[ref43] Huang W. Fang X. Wang H. Chen F. Duan H. Bi Y.. (2018). Biosynthesis of AgNPs by *B. maydis* and its antifungal effect against *Exserohilum turcicum*. IET Nanobiotechnol. 12, 585–590. doi: 10.1049/iet-nbt.2017.0263, PMID: 30095417 PMC8676670

[ref45] Iwu C. D. Okoh A. I. (2019). Preharvest transmission routes of fresh produce associated bacterial pathogens with outbreak potentials: a review. Int. J. Environ. Res. Public Health 16:4407. doi: 10.3390/ijerph16224407, PMID: 31717976 PMC6888529

[ref46] Jain N. Bhargava A. Majumdar S. Tarafdar J. C. Panwar J. (2011). Extracellular biosynthesis and characterization of silver nanoparticles using *Aspergillus flavus* NJP08: a mechanism perspective. Nanoscale 3, 635–641. doi: 10.1039/C0NR00656D, PMID: 21088776

[ref47] Johnson K. B. Stockwell V. O. (1998). Management of fire blight: a case study in microbial ecology. Annu. Rev. Phytopathol. 36, 227–248. doi: 10.1146/annurev.phyto.36.1.227 15012499

[ref48] Joshi P. A. Bonde S. R. Gaikwad S. C. Gade A. K. Abd-Elsalam K. Rai M. K. (2013). Comparative studies on synthesis of silver nanoparticles by *Fusarium oxysporum* and *Macrophomina phaseolina* and its efficacy against bacteria and *Malassezia furfur*. J. Bionanosci. 7, 378–385. doi: 10.1166/jbns.2013.1148

[ref50] Lahiri D. Nag M. Ray R. R. (2021). “Mycosynthesis of silver nanoparticles: mechanism and applications” in Nanobiotechnology—Microbes and Plant Assisted Synthesis of Nanoparticles, Mechanisms and Applications. eds. Ghosh S. Webster T. (Netherlands: Elsevier), 91–104.

[ref51] Larsson D. G. J. Flach C. F. (2022). Antibiotic resistance in the environment. Nat. Rev. Microbiol. 20, 257–269. doi: 10.1038/s41579-021-00649-x, PMID: 34737424 PMC8567979

[ref52] Li G. He D. Qian Y. Guan B. Gao S. Cui Y.. (2012). Fungus-mediated green synthesis of silver nanoparticles using *Aspergillus terreus*. Int. J. Mol. Sci. 13, 466–476. doi: 10.3390/ijms13010466, PMID: 22312264 PMC3269698

[ref53] Li W. R. Xie X. B. Shi Q. S. Zeng H. Y. Ou-Yang Y. S. Chen Y. B. (2010). Antibacterial activity and mechanism of silver nanoparticles on *Escherichia coli*. Appl. Microbiol. Biotechnol. 85, 1115–1122. doi: 10.1007/s00253-009-2159-5 19669753

[ref54] Magaldi S. Mata-Essayag S. Hartung de Capriles C. Perez C. Colella M. T. Olaizola C.. (2004). Well diffusion for antifungal susceptibility testing. Int. J. Infect. Dis. 8, 39–45. doi: 10.1016/j.ijid.2003.03.002 14690779

[ref55] Majeed S. Danish M. Zahrudin A. H. B. Dash G. K. (2018). Biosynthesis and characterization of silver nanoparticles from fungal species and its antibacterial and anticancer effect. Karbala Int. J. Mod. Sci. 4, 86–92. doi: 10.1016/j.kijoms.2017.11.002

[ref56] Maji A. Nath R. (2015). Pathogenecity test by using different inoculation methods on *Xanthomonas campestris* pv. *campestris* caused of black rot of cabbage. Int. J. Appl. Res. Nat. Prod. 3, 53–58.

[ref57] Malandrakis A. A. Kavroulakis N. Chrysikopoulos C. V. (2019). Use of copper, silver and zinc nanoparticles against foliar and soil-borne plant pathogens. Sci. Total Environ. 670, 292–299. doi: 10.1016/j.scitotenv.2019.03.210, PMID: 30903901

[ref58] Mansfield J. Genin S. Magori S. Citovsky V. Sriariyanum M. Ronald P.. (2012). Top 10 plant pathogenic bacteria in molecular plant pathology. Mol. Plant Pathol. 13, 614–629. doi: 10.1111/j.1364-3703.2012.00804.x, PMID: 22672649 PMC6638704

[ref59] Markowska K. Grudniak A. M. Wolska K. I. (2013). Silver nanoparticles as an alternative strategy against bacterial biofilms. Acta Biochim. Pol. 60, 523–530. doi: 10.18388/abp.2013_2016 24432308

[ref60] Martinez-Gutierrez F. Boegli L. Agostinho A. Sánchez E. M. Bach H. Ruiz F.. (2013). Anti-biofilm activity of silver nanoparticles against different microorganisms. Biofouling 29, 651–660. doi: 10.1080/08927014.2013.794225, PMID: 23731460

[ref61] Mistry H. Thakor R. Patil C. Trivedi J. Bariya H. (2021). Biogenically proficient synthesis and characterization of silver nanoparticles employing marine procured fungi *Aspergillus brunneoviolaceus* along with their antibacterial and antioxidative potency. Biotechnol. Lett. 43, 307–316. doi: 10.1080/08927014.2013.794225, PMID: 32944816

[ref02] Muthulakshmi L. Suganya K. Murugan M. Annaraj J. Duraipandiyan V. Al Farraj D. A.. (2022). Antibiofilm efficacy of novel biogenic silver nanoparticles from *Terminalia catappa* against food-borne *Listeria monocytogenes* ATCC 15,313 and mechanisms investigation *in-vivo* and *in-vitro*. J. King Saud Univ. Sci. 34:102083. doi: 10.1016/j.jksus.2022.102083

[ref62] Nawaz A. Tariq J. A. Lodhi A. M. Memon R. M. (2020). Studies on characteristics of *Xanthomonas oryzae* isolates associated with Rice crop. J. App. Res. Plant Sci. 1, 30–35. doi: 10.38211/joarps.2020.1.1.5

[ref63] Olfati A. Kahrizi D. Balaky S. T. J. Sharifi R. Tahir M. B. Darvishi E. (2021). Green synthesis of nanoparticles using *Calendula officinalis* extract from silver sulfate and their antibacterial effects on *Pectobacterium caratovorum*. Inorg. Chem. Commun. 125:108439. doi: 10.1016/j.inoche.2020.108439

[ref64] Olivares E. Badel-Berchoux S. Provot C. Prévost G. Bernardi T. Jehl F. (2020). Clinical impact of antibiotics for the treatment of *Pseudomonas aeruginosa* biofilm infections. Front. Microbiol. 10:2894. doi: 10.3389/fmicb.2019.02894, PMID: 31998248 PMC6962142

[ref65] Osorio-Echavarría J. Osorio-Echavarría J. Ossa-Orozco C. P. Gómez-Vanegas N. A. (2021). Synthesis of silver nanoparticles using white-rot fungus Anamorphous *Bjerkandera* sp. R1: influence of silver nitrate concentration and fungus growth time. Sci. Rep. 11, 1–14. doi: 10.1038/s41598-021-82514-8, PMID: 33589657 PMC7884706

[ref66] Panáček A. Smékalová M. Kilianová M. Prucek R. Bogdanová K. Večeřová R.. (2016). Strong and nonspecific synergistic antibacterial efficiency of antibiotics combined with silver nanoparticles at very low concentrations showing no cytotoxic effect. Molecules 21:26. doi: 10.3390/molecules21010026, PMID: 26729075 PMC6273824

[ref68] Peeters E. Nelis H. J. Coenye T. (2008). Comparison of multiple methods for quantification of microbial biofilms grown in microtiter plates. J. Microbiol. Methods 72, 157–165. doi: 10.1016/j.mimet.2007.11.010, PMID: 18155789

[ref69] Peil A. Emeriewen O. F. Khan A. Kostick S. Malnoy M. (2021). Status of fire blight resistance breeding in Malus. J. Plant Pathol. 103, 3–12. doi: 10.1007/s42161-020-00581-8

[ref70] Pérombelon M. C. M. (2002). Potato diseases caused by soft rot erwinias: an overview of pathogenesis. Plant Pathol. 51, 1–12. doi: 10.1046/j.0032-0862.2001.Shorttitle.doc.x

[ref71] Pérombelon M. C. M. Kelman A. (1980). Ecology of the soft rot erwinias. Annu. Rev. Phytopathol. 18, 361–387. doi: 10.1146/annurev.py.18.090180.002045

[ref72] Qing Y. Cheng L. Li R. Liu G. Zhang Y. Tang X.. (2018). Potential antibacterial mechanism of silver nanoparticles and the optimization of orthopedic implants by advanced modification technologies. Int. J. Nanomedicine 13, 3311–3327. doi: 10.2147/IJN.S165125, PMID: 29892194 PMC5993028

[ref73] Rai M. Bonde S. Golinska P. Trzcińska-Wencel J. Gade A. Abd-Elsalam K. A.. (2021a). *Fusarium* as a novel fungus for the synthesis of nanoparticles: mechanism and applications. J. Fungi 7:139. doi: 10.3390/jof7020139, PMID: 33672011 PMC7919287

[ref74] Rai M. Ingle A. P. Trzcińska-Wencel J. Wypij M. Bonde S. Yadav A.. (2021b). Biogenic silver nanoparticles: what we know and what do we need to know? Nano 11:2901. doi: 10.3390/nano11112901, PMID: 34835665 PMC8624974

[ref75] Ramachandran R. Sangeetha D. (2017). Antibiofilm efficacy of silver nanoparticles against biofilm forming multidrug resistant clinical isolates. J. Pharm. Innov. 6:36.

[ref76] Rogowska A. Rafińska K. Pomastowski P. Walczak J. Railean-Plugaru V. Buszewska-Forajta M.. (2017). Silver nanoparticles functionalized with ampicillin. Electrophoresis 38, 2757–2764. doi: 10.1002/elps.201700093 28704596

[ref78] Ruden S. Hilpert K. Berditsch M. Wadhwani P. Ulrich A. S. (2009). Synergistic interaction between silver nanoparticles and membrane-permeabilizing antimicrobial peptides. Antimicrob. Agents Chemother. 53, 3538–3540. doi: 10.1128/AAC.01106-08, PMID: 19528287 PMC2715642

[ref79] Sahoo J. P. Mishra A. P. Samal K. C. Dash A. K. (2021). Insights into the antibiotic resistance in biofilms–a review. Environ. Conserv. J. 22, 59–67. doi: 10.36953/ECJ.2021.22307

[ref80] Salem S. S. (2022). Baker’s yeast-mediated silver nanoparticles: characterisation and antimicrobial biogenic tool for suppressing pathogenic microbes. BioNanoScience 12, 1220–1229. doi: 10.1007/s12668-022-01026-5

[ref81] Salem S. S. Ali O. M. Reyad A. M. Abd-Elsalam K. A. Hashem A. H. (2022). Pseudomonas indica-mediated silver nanoparticles: antifungal and antioxidant biogenic tool for suppressing mucormycosis fungi. J. Fungi 8:126. doi: 10.3390/jof8020126, PMID: 35205879 PMC8874487

[ref82] Salem S. S. Hammad E. N. Mohamed A. A. El-Dougdoug W. (2023). A comprehensive review of nanomaterials: types, synthesis, characterization, and applications. Biointerface Res. Appl. Chem. 13:41. doi: 10.33263/BRIAC131.041

[ref83] Salleh A. Naomi R. Utami N. D. Mohammad A. W. Mahmoudi E. Mustafa N.. (2020). The potential of silver nanoparticles for antiviral and antibacterial applications: a mechanism of action. Nano 10:1566. doi: 10.3390/nano10081566, PMID: 32784939 PMC7466543

[ref84] Salvioni L. Galbiati E. Collico V. Alessio G. Avvakumova S. Corsi F.. (2017). Negatively charged silver nanoparticles with potent antibacterial activity and reduced toxicity for pharmaceutical preparations. Int. J. Nanomedicine 12, 2517–2530. doi: 10.2147/IJN.S127799, PMID: 28408822 PMC5383075

[ref85] Scallan Walter E. J. Griffin P. M. Bruce B. B. Hoekstra R. M. (2021). Estimating the number of illnesses caused by agents transmitted commonly through food: a scoping review. Foodborne Pathog. Dis. 18, 841–858. doi: 10.1089/fpd.2021.0038, PMID: 34529512

[ref86] Schaad N. W. Sitterly W. R. Humaydan H. (1980). Relationship of incidence of seedborne *Xanthomonas campestris* to black rot of crucifers. Plant Dis. 64, 91–92. doi: 10.1094/PD-64-91

[ref87] Shaheen T. I. Salem S. S. Fouda A. (2021). “Current advances in fungal nanobiotechnology: Mycofabrication and applications” in Microbial Nanobiotechnology: Principles and Applications. eds. Lateef A. Gueguim-Kana E. B. Dasgupta N. Ranjan S. (Singapore: Springer)

[ref88] Shenge K. C. Mabagala R. B. Mortensen C. N. Stephan D. Wydra K. (2007). First report of bacterial speck of tomato caused by *Pseudomonas syringae* pv. tomato in Tanzania. Plant Dis. 91:462. doi: 10.1094/PDIS-91-4-0462C, PMID: 30781201

[ref89] Smitha S. L. Nissamudeen K. M. Philip D. Gopchandran K. G. (2008). Studies on surface plasmon resonance and photoluminescence of silver nanoparticles. Spectrochim. Acta A Mol. Biomol. Spectrosc. 71, 186–190. doi: 10.1016/j.saa.2007.12.002, PMID: 18222106

[ref90] Soliman M. K. Salem S. S. Abu-Elghait M. Azab M. S. (2022). Biosynthesis of silver and gold nanoparticles and their efficacy towards antibacterial, antibiofilm, cytotoxicity, and antioxidant activities. Appl. Biochem. Biotechnol. 195, 1158–1183. doi: 10.1007/s12010-022-04199-7, PMID: 36342621 PMC9852169

[ref91] Sundin G. W. Castiblanco L. F. Yuan X. Zeng Q. Yang C. H. (2016). Bacterial disease management: challenges, experience, innovation and future prospects: challenges in bacterial molecular plant pathology. Mol. Plant Pathol. 17, 1506–1518. doi: 10.1111/mpp.12436, PMID: 27238249 PMC6638406

[ref03] Tariq M. Mohammad K. N. Ahmed B. Siddiqui M. A. Lee J. (2022). Biological synthesis of silver nanoparticles and prospects in plant disease management. Molecules 27:4754. doi: 10.3390/molecules27154754 35897928 PMC9330430

[ref04] Tian X. Jiang X. Welch C. Croley T. R. Wong T. Y. Chen C.. (2018). Bactericidal effects of silver nanoparticles on lactobacilli and the underlying mechanism. ACS Appl. Mater. Interfaces 10, 8443–8450 doi: 10.1021/acsami.7b17274 29481051

[ref92] Tomah A. A. Alamer I. S. A. Li B. Zhang J. Z. (2020). Mycosynthesis of silver nanoparticles using screened *Trichoderma* isolates and their antifungal activity against *Sclerotinia sclerotiorum*. Nano 10:1955. doi: 10.3390/nano10101955, PMID: 33008115 PMC7599925

[ref93] Tomaszewska E. Soliwoda K. Kadziola K. Tkacz-Szczesna B. Celichowski G. Cichomski M.. (2013). Detection limits of DLS and UV-Vis spectroscopy in characterization of polydisperse nanoparticles colloids. J. Nanomat. 2013, 1–10. doi: 10.1155/2013/313081

[ref94] Vanneste J. (2000). Fire Blight: The Disease and Its Causative Agent, Erwinia Amylovora. New York, USA: CABI

[ref95] Vigneshwaran N. Ashtaputre N. M. Varadarajan P. V. Nachane R. P. Paralikar K. M. Balasubramanya R. H. (2007). Biological synthesis of silver nanoparticles using the fungus *Aspergillus flavus*. Mater. Lett. 61, 1413–1418. doi: 10.1016/j.matlet.2006.07.042

[ref96] Wang L. Hu C. Shao L. (2017). The antimicrobial activity of nanoparticles: present situation and prospects for the future. Int. J. Nanomedicine 12, 1227–1249. doi: 10.2147/IJN.S121956, PMID: 28243086 PMC5317269

[ref97] Wang C. Xu S. Zhang K. Li M. Li Q. Xiao R.. (2017). Streptomycin-modified Fe3O4–Au@ Ag core–satellite magnetic nanoparticles as an effective antibacterial agent. J. Mater. Sci. 52, 1357–1368. doi: 10.1007/s10853-016-0430-6

[ref98] White T. J. Bruns T. Lee S. J. W. T. Taylor J. (1990). Amplification and direct sequencing of fungal ribosomal RNA genes for phylogenetics. PCR Protocols 18, 315–322. doi: 10.1016/B978-0-12-372180-8.50042-1

[ref99] Wigginton N. S. Titta A. D. Piccapietra F. Dobias J. A. N. Nesatyy V. J. Suter M. J.. (2010). Binding of silver nanoparticles to bacterial proteins depends on surface modifications and inhibits enzymatic activity. Environ. Sci. Technol. 44, 2163–2168. doi: 10.1021/es903187s, PMID: 20158230

[ref100] World Health Organization (WHO). (2021). Antimicrobial Resistance. Available at: https://www.who.int/news-room/fact-sheets/detail/antimicrobial-resistance (Accessed May 15, 2022).

[ref101] Wypij M. Czarnecka J. Świecimska M. Dahm H. Rai M. Golinska P. (2018). Synthesis, characterization and evaluation of antimicrobial and cytotoxic activities of biogenic silver nanoparticles synthesized from *Streptomyces xinghaiensis* OF1 strain. World J. Microbiol. Biotechnol. 34, 23–13. doi: 10.1007/s11274-017-2406-3, PMID: 29305718 PMC5756267

[ref102] Wypij M. Jędrzejewski T. Ostrowski M. Trzcińska J. Rai M. Golińska P. (2020). Biogenic silver nanoparticles: assessment of their cytotoxicity, genotoxicity and study of capping proteins. Molecules 25:3022. doi: 10.3390/molecules25133022, PMID: 32630696 PMC7412363

[ref103] Wypij M. Jędrzejewski T. Trzcińska-Wencel J. Ostrowski M. Rai M. Golińska P. (2021). Green synthesized silver nanoparticles: antibacterial and anticancer activities, biocompatibility, and analyses of surface-attached proteins. Front. Microbiol. 12:632505. doi: 10.3389/fmicb.2021.632505, PMID: 33967977 PMC8100210

[ref104] Wypij M. Ostrowski M. Piska K. Wójcik-Pszczoła K. Pękala E. Rai M.. (2022). Novel antibacterial, cytotoxic and catalytic activities of silver nanoparticles synthesized from acidophilic actinobacterial SL19 with evidence for protein as coating biomolecule. J. Microbiol. Biotechnol. 32, 1195–1208. doi: 10.4014/jmb.2205.05006, PMID: 36116918 PMC9628977

[ref105] Wypij M. Trzcińska-Wencel J. Golińska P. Avila-Quezada G. D. Ingle A. P. Rai M. (2023). The strategic applications of natural polymer nanocomposites in food packaging and agriculture: chances, challenges, and consumers’ perception. Front. Chem. 10:1106230. doi: 10.3389/fchem.2022.1106230, PMID: 36704616 PMC9871319

[ref106] Xie W. Y. Shen Q. Zhao F. J. (2018). Antibiotics and antibiotic resistance from animal manures to soil: a review. Eur. J. Soil Sci. 69, 181–195. doi: 10.1111/ejss.12494

[ref107] Yuan Y. G. Peng Q. L. Gurunathan S. (2017). Effects of silver nanoparticles on multiple drug-resistant strains of *Staphylococcus aureus* and *Pseudomonas aeruginosa* from mastitis-infected goats: an alternative approach for antimicrobial therapy. Int. J. Mol. Sci. 18:569. doi: 10.3390/ijms18030569, PMID: 28272303 PMC5372585

[ref108] Zhao X. Zhou L. Riaz Rajoka M. S. Yan L. Jiang C. Shao D.. (2018). Fungal silver nanoparticles: synthesis, application and challenges. Crit. Rev. Biotechnol. 38, 817–835. doi: 10.1080/07388551.2017.1414141 29254388

[ref109] Zhu Q. Gooneratne R. Hussain M. A. (2017). *Listeria monocytogenes* in fresh produce: outbreaks, prevalence and contamination levels. Foods 6:21. doi: 10.3390/foods6030021, PMID: 28282938 PMC5368540

