# Supplementary material for: Biogenic nanosilver bearing antimicrobial and antibiofilm activities and its potential for application in agriculture and industry
Source: Front Microbiol. 2023 Feb 20;14:1125685. doi: 10.3389/fmicb.2023.1125685 (PMC9986290; doi:10.3389/fmicb.2023.1125685)
Supplement: Supplementary file 1 [file Data_Sheet_1.pdf]

Supplementary Material

Biogenic nanosilver bearing antimicrobial and antibiofilm activities and its potential for application in agriculture and industry

Joanna Trzcińska-Wencel [1*], Magdalena Wypij [1*], Mahendra Rai [1,2], Patrycja Golińska[1]

[1]Department of Microbiology, Nicolaus Copernicus University in Toruń, Toruń, Poland;

[2]Nanobiotechnology Laboratory, Department of Biotechnology, SGB Amravati University, Amravati, India

*** Correspondence:**
Joanna Trzcińska-Wencel and Magdalena Wypij
trzcinska@doktorant.umk.pl and mwypij@umk.pl

Supplementary figures:

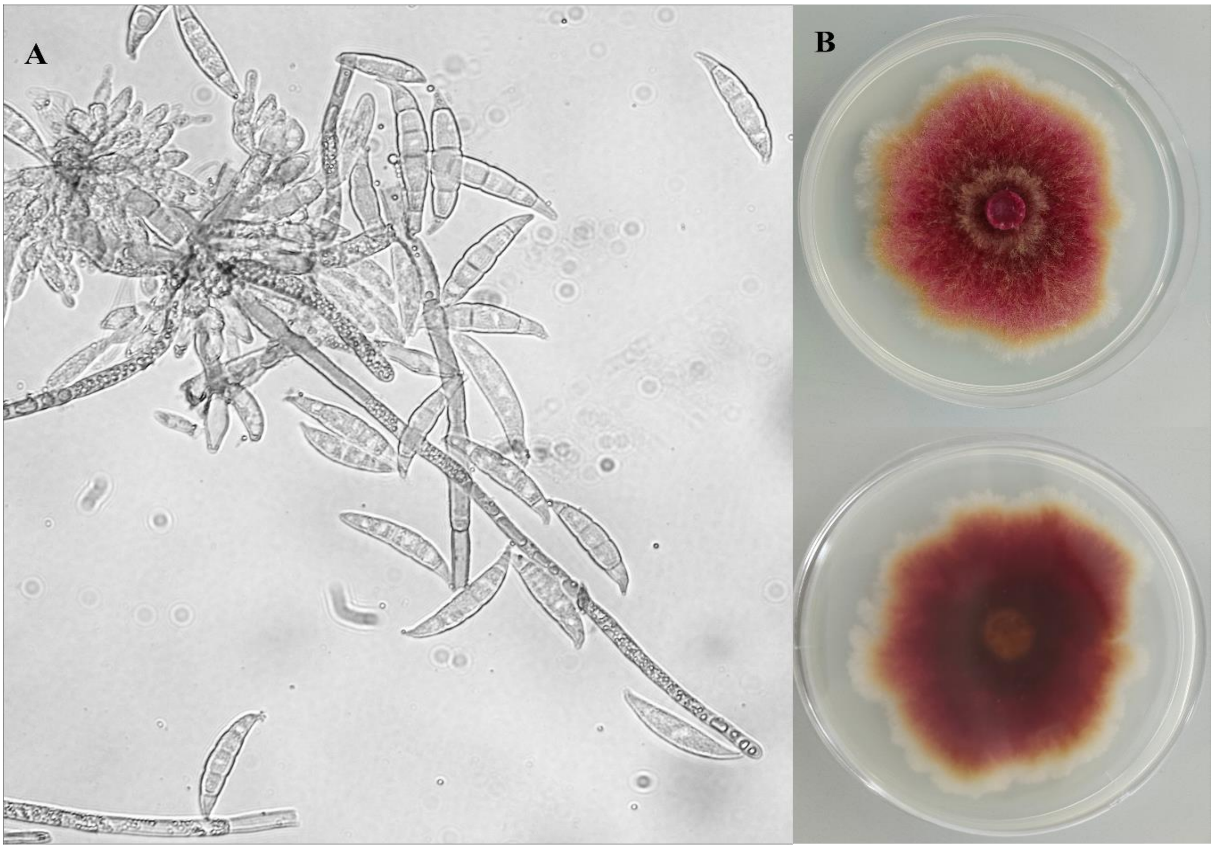

Supplementary Figure S1. Fungal strain *Fusarium culmorum* strain JTW1: light microscopic observation (A), growth on the potato dextrose agar (B).

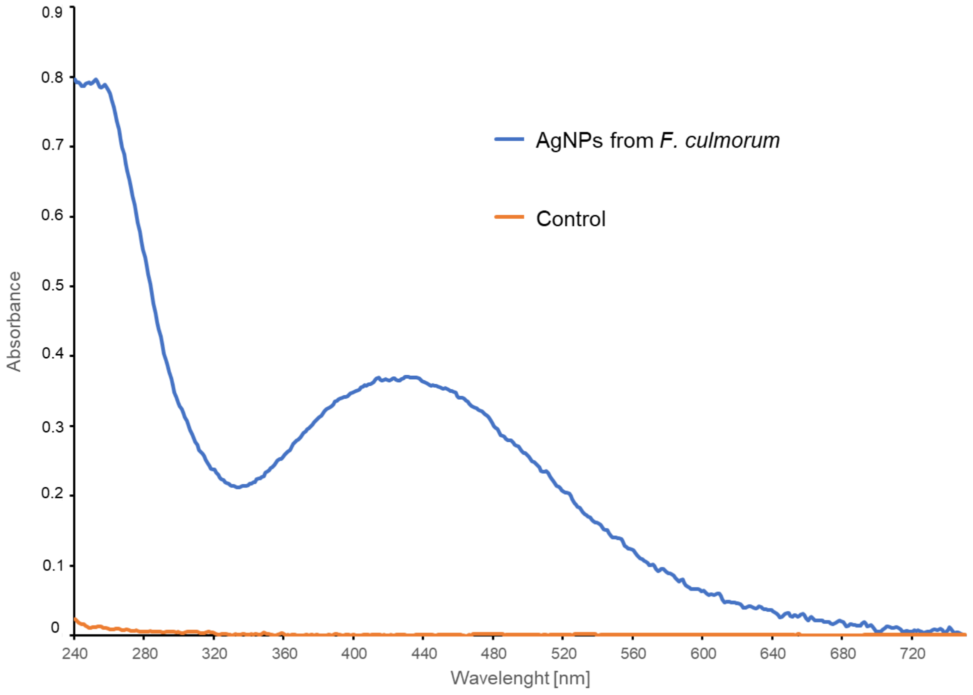

Supplementary Figure S2. The UV-Visible spectra of AgNPs from *Fusarium culmorum* strain JTW1 with the maximum absorbance peak at 430 nm.

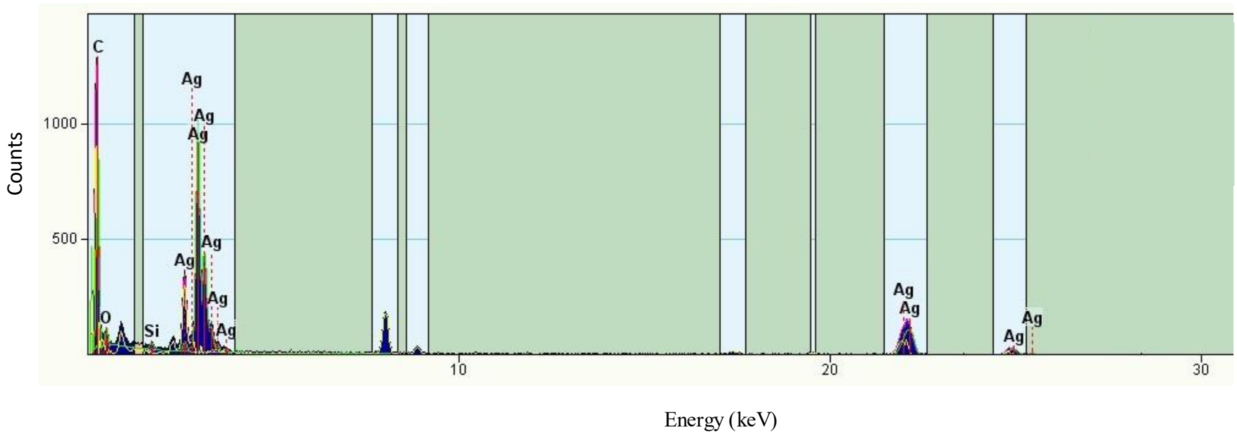

Supplementary Figure S3. Energy dispersive X-ray (EDX) spectrum of AgNPs from *Fusarium culmorum* strain JTW1.

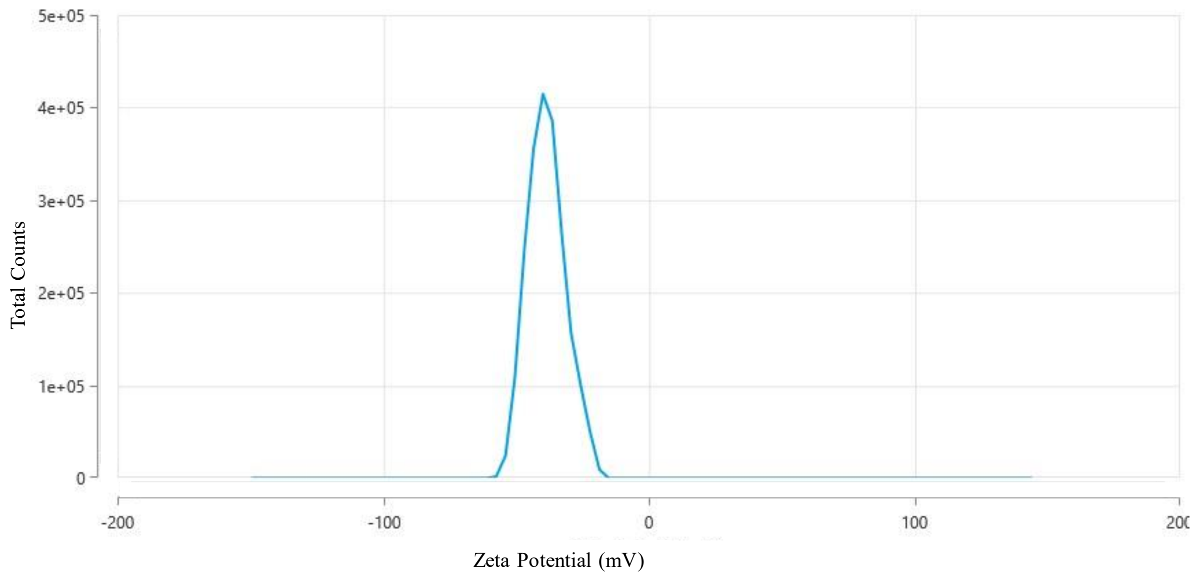

Supplementary Figure S4. The zeta potential of AgNPs from *Fusarium culmorum* strain JTW1.

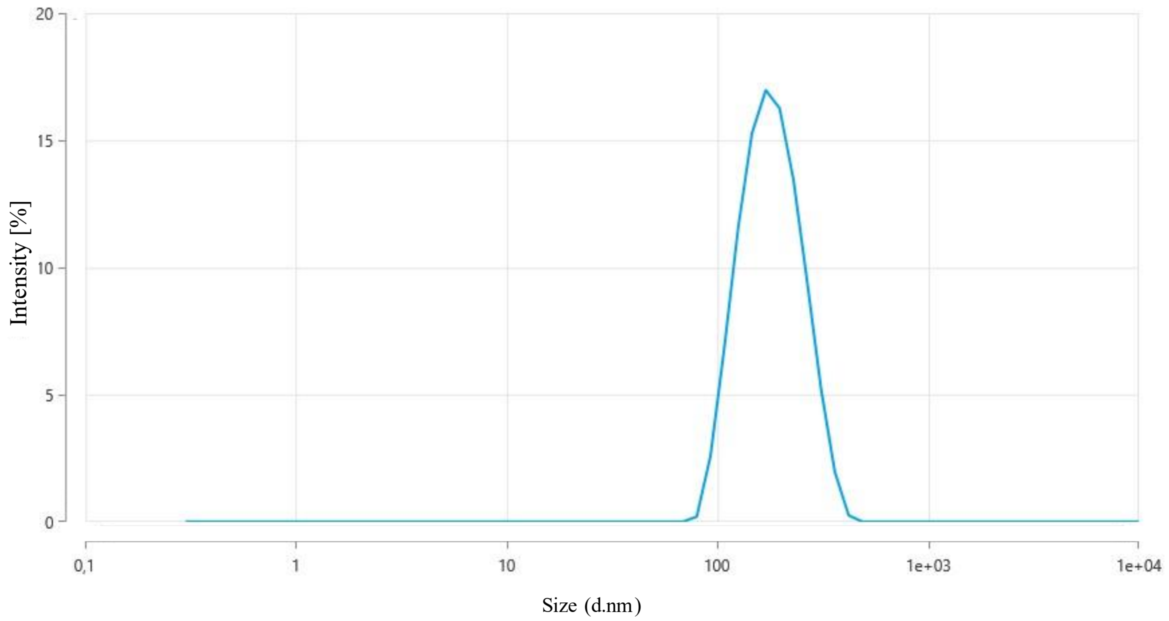

Supplementary Figure S5. Particle size distribution of AgNPs from *Fusarium culmorum* strain JTW1 estimated using dynamic light scattering (DLS).

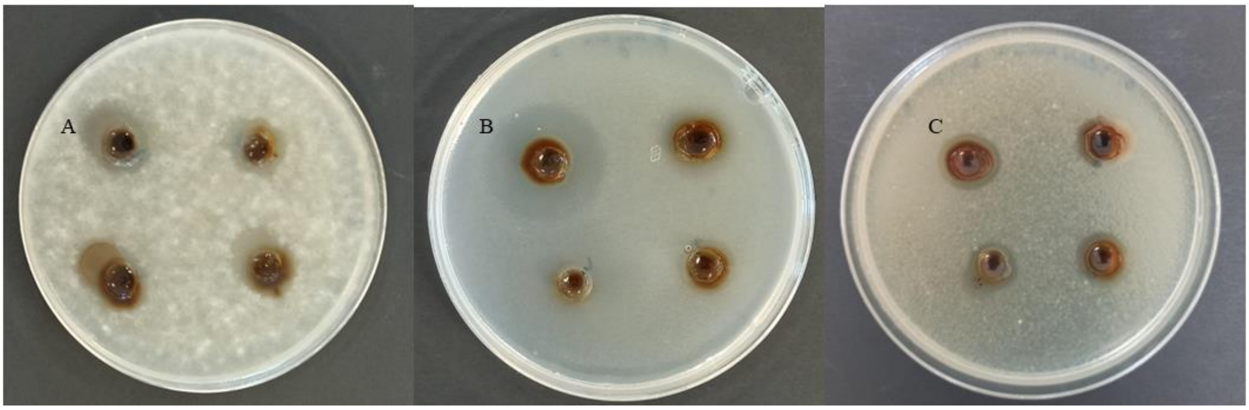

Supplementary Figure S6. Antifungal activity of AgNPs from *Fusarium culmorum* strain JTW1 against *Botrytis cinerea* IOR 1873 (A), *Phoma lingam* IOR 2284 (B) and *Sclerotinia sclerotiorum* IOR 2242 (C).
